# Lewis Pair‐Engineered CuMnO_x_ as Cold‐Adapted Multinanozyme for Cooperative Hydrolytic and Oxidative Degradation of Raw Corn Stalk

**DOI:** 10.1002/advs.202519235

**Published:** 2026-02-15

**Authors:** Huile Liu, Ziyi Di, Qing Tian, Yue Zhou, Rong Yang, Zhiyi Bai, Haoyu Wang, Yao Chen, Lianbing Zhang

**Affiliations:** ^1^ School of Life Sciences and Technology Northwestern Polytechnical University Xi'an China; ^2^ Nanozyme Laboratory in Zhongyuan Henan Academy of Innovations in Medical Science Zhengzhou China

**Keywords:** cold‐adapted nanozyme, full lignocellulose utilization, glycosidase, oxidase, polysaccharides degradation

## Abstract

The full‐component utilization of lignocellulosic biomass under mild conditions remains a formidable challenge for both biocatalytic systems and industrial processes. Herein, we report a Lewis pair engineering strategy to construct a defective Cu‐doped Mn oxide nanozyme (D‐CuMnO_x_) featuring the simultaneous introduction of manganese and oxygen vacancies. The resulting undercoordinated Mn sites act as Lewis acids to activate glycosidic bonds, while adjacent oxygen species serve as Lewis bases to promote nucleophilic attack and electron transfer, thereby collectively lowering the energy barriers for both hydrolytic and oxidative reactions. As a consequence, D‐CuMnO_x_ exhibits an approximately fourfold enhancement in glycosidase activity and markedly improved cold‐adapted performance compared with oxygen‐vacancy‐only CuMnO_x_, while simultaneously maintaining robust oxidase‐like activity. Benefiting from these advantages, D‐CuMnO_x_ serves as a cooperative hydrolytic‐oxidative platform that enables the depolymerization of cellulose and hemicellulose alongside the oxidative cleavage of lignin, thereby achieving simultaneous degradation of the major components in raw corn stalks under mild and low‐temperature conditions. This work establishes Lewis pair engineering as a versatile strategy for the rational design of multifunctional cold‐adapted nanozymes and highlights their considerable potential for sustainable biomass valorization.

## Graphical abstract

A Lewis pair site design strategy was established to construct Cu‐doped Mn‐based amorphous nanozymes, where Mn and oxygen vacancies generate cooperative acid‐base pairs. The resulting multinanozyme integrates glycosidase‐, oxidase‐, and cold‐adapted enzyme‐mimicking activities, enabling cooperative depolymerization of cellulose and hemicellulose alongside oxidative disassembly of lignin, thus achieving full‐component degradation of raw corn stalk under mild, low‐temperature conditions.

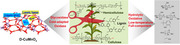

## 1 Introduction

As the most abundant renewable carbon resource on earth, lignocellulose can be converted into biofuels, value‐added chemicals, and biobased materials, providing a promising alternative to fossil feedstocks [1, 2, 3, 4]. However, its highly cross‐linked architecture, composed of cellulose microfibrils embedded within hemicellulose and lignin matrices, renders it intrinsically recalcitrant to degradation, particularity with respect to full‐component utilization. Conventional strategies rely on microbial consortia or cocktails of hydrolytic and oxidative enzymes to achieve efficient conversion [5, 6]. However, these approaches are challenged by their instability, high cost of batch production, and particularly low efficiency at low temperatures. These findings underscore the urgent need for robust catalytic platforms for efficient biomass utilization under mild conditions.

To address these challenges, nanozymes have emerged as a promising class of artificial catalysts that combine high activity with operational stability, even under harsh conditions [7, 8]. Advances in rational design have yielded nanozymes with catalytic performance comparable to, and in some cases exceeding, their natural counterparts [9]. Of particular note, our group recently reported the first cold‐adapted nanozyme capable of maintaining stable activity across 0–45°C, extending beyond the operating temperature limits of enzymes and underscoring nanozymes as compelling candidates for next‐generation biocatalysis [10]. Nevertheless, existing efforts have rarely focused on hydrolytic nanozymes, particularly those with cold‐adapted properties, and multi‐family systems that simultaneously couple hydrolytic and oxidative functions.

Hydrolytic nanozymes developed to date are mainly based on Zn‐ or Ce‐containing nanomaterials [11, 12, 13, 14], which mimic the cofactors of natural glycoside hydrolases (GHs). In fact, manganese (Mn) also serves as a critical cofactor in certain GHs, where it stabilizes transition states and facilitates proton transfer during glycosidic bond cleavage [15, 16, 17]. However, systematic studies on Mn‐based hydrolases and their underlying mechanisms remain to be explored. Meanwhile, Mn‐based nanomaterials have been extensively studied for their oxidase‐like activities, particularly those simultaneously possess cold‐adapted characteristics [10, 18, 19, 20, 21]. These advances highlight Mn‐based systems as promising candidates for constructing multifunctional nanozymes that combining hydrolytic and oxidative activities while retaining cold‐adapted properties at low temperatures.

Beyond these intrinsic advantages, strategies to optimize the hydrolytic activity of Mn‐based nanozymes are highly desirable. Conventional approaches mainly relied on creating Lewis‐acidic centers that mimic enzymatic active sites [13, 22, 23]. However, their catalytic efficiency is limited by the absence of complementary proton transfer and nucleophile activation. Recent studies have demonstrated that metal defects can create under‐coordinated metal centers acting as Lewis acids, while the neighboring lattice oxygen or defect‐associated oxygen sites become electron‐rich and serve as Lewis bases. Such defect‐derived Lewis pairs are demonstrated to be highly effective in oxidation‐related processes, such as ozone decomposition and selective oxidation [24, 25, 26]. This spatial proximity of acid‐base cooperation should closely mimic the catalytic mechanism of natural GHs, thereby enabling more efficient cleavage of glycosidic bonds. Concurrently, such motifs are expected to promote oxidase‐like activity by enabling rapid electron transfer between under‐coordinated metal centers and nearby electron‐rich oxygen‐deficient sites [27, 28].

Herein, we report a defective Cu‐doped Mn‐based nanozyme (D‐CuMnO_x_) prepared via Cu doping and structural amorphization, in which the simultaneous generation of Mn vacancies (V_Mn_) and oxygen vacancies (V_O_) introduces abundant Lewis pairs. The presence and catalytic roles of these Lewis pairs were confirmed by structural characterization, catalytic evaluation, and theoretical calculations. Acid‐base pairs facilitate nucleophilic attack and protonation of glycosidic oxygen while promoting fast electron transfer. Consequently, D‐CuMnO_x_ displays greatly enhanced hydrolytic activity toward polysaccharides, alongside broad‐spectrum oxidase‐like activity for lignin degradation. Moreover, its exceptional cold adaptability ensures high catalytic efficiency under low‐temperature conditions, enabling simultaneous degradation of three major components in corn stalks under mild environmental conditions.

## 2 Results and Discussion

### 2.1 Structure and Defect Characterization

**FIGURE 1:**
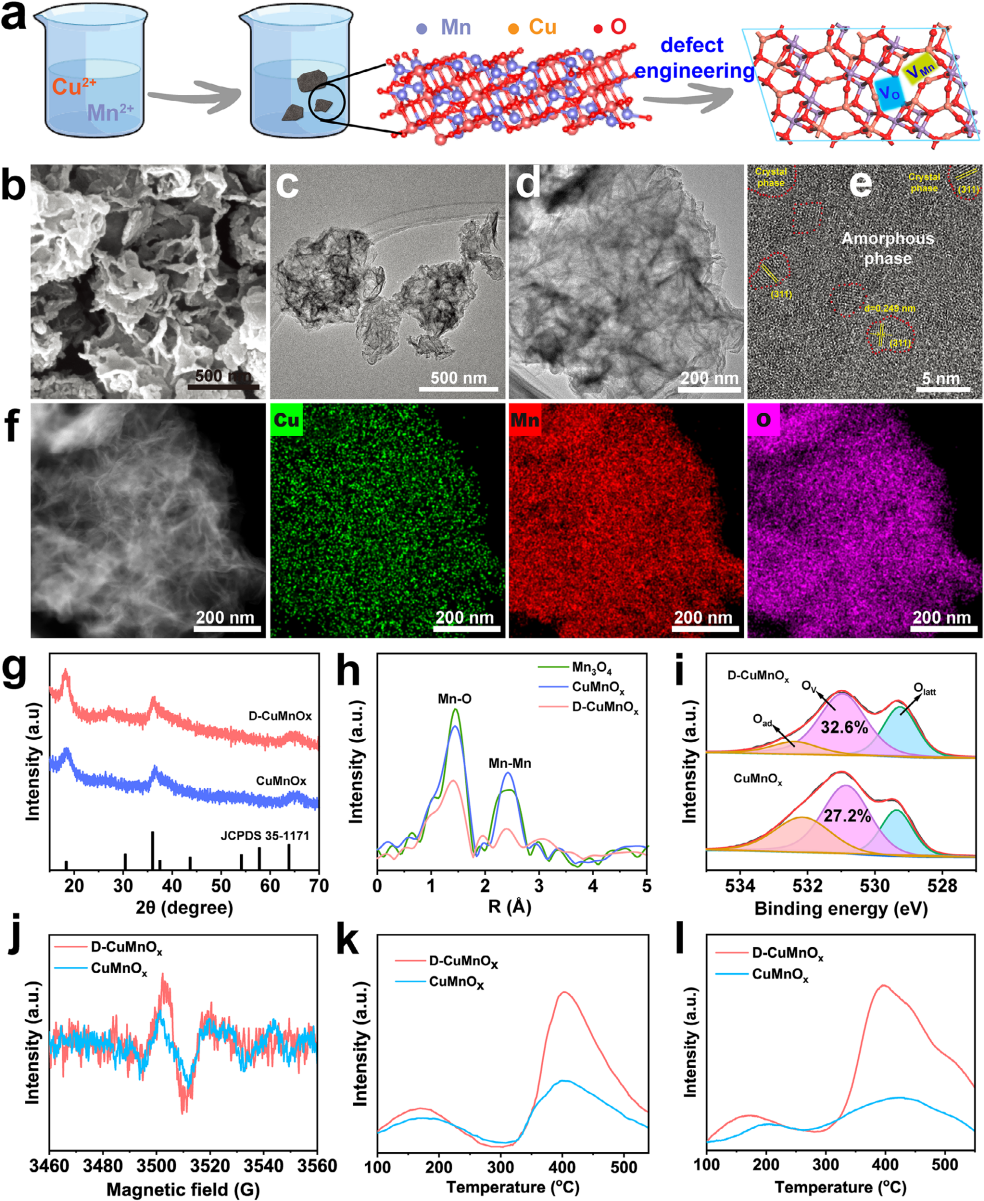
(a) Scheme of D‐CuMnOx synthesis. (b) SEM, (c) TEM, (d, e) HRTEM, and (f) HAADF images of D‐CuMnO_x_ with corresponding EDS mapping (Mn, Cu, and O). (g) XRD spectra, (h) K^3^‐FT Mn K‐edge EXAFS spectra, (i) O1s XPS spectrum, (j) EPR spectrum, (k) CO_2_‐TPD and (l) NH_3_‐TPD profiles.

The designed D‐CuMnO_x_ was synthesized by integrating Cu doping with amorphous engineering (Figure 1a), following previously reported methods [29, 30]. Scanning electron microscopy (SEM, Figure 1b) and transmission electron microscopy (TEM, Figure 1c,d) images revealed that D‐CuMnO_x_ consists of flake‐like nanosheets with lateral dimensions of ∼200–500 nm. High‐resolution TEM (Figure 1e) further revealed the coexistence of amorphous and crystalline domains within D‐CuMnO_x_. The crystalline domains displayed lattice fringes with an interplanar spacing of 0.25 nm, which can be ascribed to the (311) planes of manganese oxide. Elemental mapping by energy‐dispersion X‐ray spectroscopy (EDS) shown in Figure 1f confirms the homogeneous distribution of Cu, Mn, and O throughout the nanosheets, demonstrating the successful incorporation of Cu into the Mn oxide framework. For comparison, a sample without Mn vacancy engineering (denoted as CuMnO_x_) was also prepared. The characterization results, including TEM and HRTEM (Figure S1), indicate that CuMnO_x_ exhibits morphology and lattice structures very similar to those of D‐CuMnO_x_. Moreover, both materials exhibited comparable X‐ray diffraction (XRD) patterns (Figure 1g), which were indexed to JCPDS No. 35–1171, corresponding to the spinel MnO_x_ phase [30]. The broad and weak diffraction peaks, lacking sharp maxima, further confirm their amorphous nature and low crystallinities.

The presence of V_Mn_ and V_O_ in D‐CuMnO_x_ was then carefully examined. X‐ray absorption spectroscopy (XAS) was employed to probe the local atomic coordination of Mn in the samples. As shown in Figure 1h, the *k* ^3^‐weighted Fourier transforms (FT) of the Mn *k*‐edge EXAFS spectra for D‐CuMnO_x_ and CuMnO_x_ exhibit two characteristic peaks, assignable to Mn‐O and Mn‐Mn interactions, consistent with those of Mn_3_O_4_. The FT intensity of D‐CuMnO_x_ was markedly lower than that of CuMnO_x_. Moreover, the coordination number (CN) of Mn‐Mn coordination numbers was significantly lower than that in CuMnO_x_ and Mn_3_O_4_ (Table S1), directly indicating the loss of coordinating Mn cations and the formation of Mn vacancies. X‐ray photoelectron spectroscopy (XPS) analysis was conducted to investigate the electronic structure of the elements. As shown in Figure 1i, the deconvoluted O 1s spectra clearly reveal contributions from lattice oxygen, surface‐adsorbed oxygen, and signals attributable to oxygen vacancies. Compared with CuMnO_x_ (27.2%), D‐CuMnO_x_ displayed a higher proportion of V_O_ (32.6%), likely associated with the introduction of V_Mn_, which facilitates the migration or detachment of oxygen from the lattice. High‐resolution Mn 2p and Cu 2p spectra (Figure S2) reveal the coexistence of multiple valence states for both Mn and Cu, including Mn^2+^, Mn^3+^, Mn^4+^, Cu^+^_,_ and Cu^2+^. Notably, the presence of V_Mn_ induces a charge imbalance, which is compensated by the oxidation of neighboring Mn ions. This contributes to a positive shift in the Mn 2p binding energies and an increased proportion of Mn^3+^ and Mn^4+^ in D‐CuMnO_x_ (Table S1), which, within the mixed‐valence states, is favorable for enhancing the oxidase‐like activity. Consistent with the XPS results, electron paramagnetic resonance (EPR) provided additional evidence for the presence of V_O_, presenting a characteristic signal at 3500–3520 G (g≈2.004) typically assigned to unpaired electrons at V_O_ (Figure 1j). The signal in D‐CuMnO_x_ was stronger than that in CuMnO_x_, indicating a higher concentration of V_O_. Moreover, due to the presence of Mn vacancies, the inductively coupled plasma (ICP) analysis (Table S1) indicates that D‐CuMnO_x_ exhibits a noticeably lower Mn atomic ratio compared with CuMnO_x_. These above‐mentioned characterizations strongly support the coexistence of V_Mn_ and V_O_ in D‐CuMnO_x_, which is expected to generate Lewis pairs.

To validate this, temperature‐programmed desorption measurements using NH_3_ (NH_3_‐TPD) and CO_2_ (CO_2_‐TPD) as probes were performed. The NH_3_‐TPD spectrum (Figure 1k) of D‐CuMnO_x_ displays two desorption peaks at ∼150°C and above 400°C, corresponding to weak and medium Lewis acid sites, respectively. Although CuMnO_x_ displayed a similar profile, its peaks were markedly weaker and shifted to lower temperatures, indicating fewer defects and reduced acidity. Similarly, the CO_2_‐TPD (Figure 1l) profile of D‐CuMnO_x_ shows two desorption signals assigned to weak and medium Lewis basic sites. In contrast, these features are almost absent in CuMnO_x_, confirming the lack of Lewis basicity and indicating that V_O_ alone cannot induce Lewis pairs. Taken together, these findings demonstrate that D‐CuMnO_x_ simultaneously accommodates both V_Mn_ and V_O_, generating Lewis pair sites within the material.

### 2.2 Cold‐Adapted GHs‐Like Activity of D‐CuMnO_x_

**FIGURE 2:**
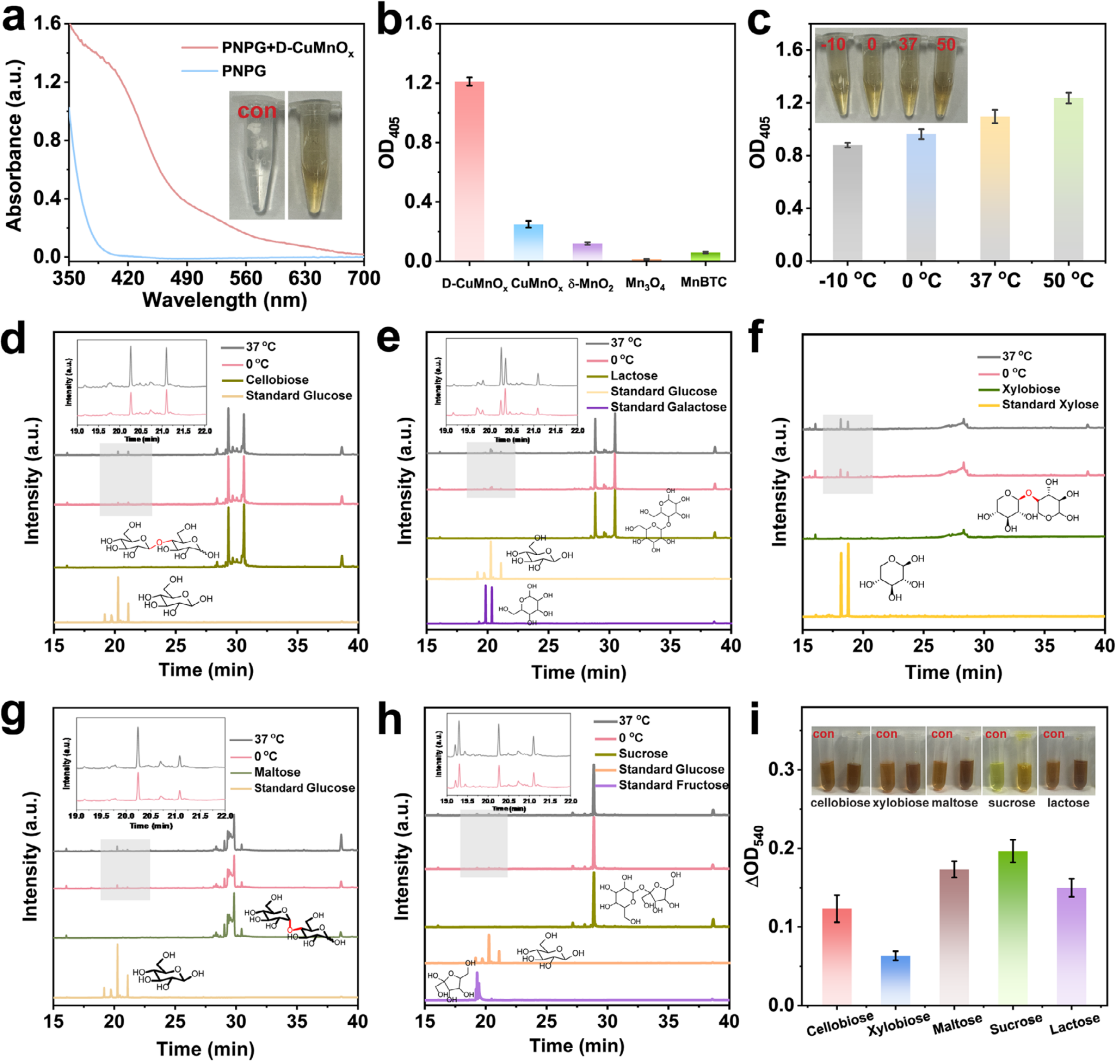
GHs‐like activity of D‐CuMnO_x_. (a) UV–vis spectra for pNPG hydrolysis, (b) comparison of activity with other Mn‐based nanozymes, and (c) activity at different temperatures. GHs‐like activity toward various disaccharides, including (d) cellobiose, (e) lactose, (f) xylobiose, (g) maltose, and (h) sucrose, with the corresponding reducing sugar concentrations quantified by the DNS method (i).

Motivated by the specific Lewis pair structure reminiscent of natural hydrolases, we investigated the GHs‐like activity of D‐CuMnO_x_ using a well‐established colorimetric assay with p‐nitrophenyl‐*β*‐D‐glucopyranoside (pNPG) as a model substrate. As shown in Figure 2a, D‐CuMnO_x_ exhibited a pronounced ability to cleave the glycosidic bond in pNPG, releasing 4‐nitrophenol (4‐NP), which has a characteristic UV–vis absorption band at 405 nm and induces a visible color change in the reaction system from colorless to yellow. Importantly, D‐CuMnO_x_ not only exhibited an approximately 4‐fold enhancement in catalytic activity compared to CuMnO_x_, but also outperformed our previously developed Mn‐based metal‐organic framework (MnBTC) as well as conventional MnO_x_ (δ‐MnO_2_ and Mn_3_O_4_) [18] (Figure 2b). Strikingly, the D‐CuMnO_x_ also presented excellent cold‐adapted performance, retaining approximately 72.2% of its catalytic efficiency as temperature decreased from 50°C to −10°C (Figure 2c). To quantitatively rationalize this behavior from an enzymatic kinetics perspective, temperature‐dependent kinetic analysis was conducted at 37°C and 0°C. As shown in Table S2, D‐CuMnO_x_ displays comparable *K*_m_ values at both temperatures, indicating that substrate affinity is largely preserved under cold conditions, while *K*_cat_ and *K*_cat_/*K*_m_ remain at relatively high levels, reflecting sustained turnover frequency and catalytic efficiency at low temperature. To the best of our knowledge, such cold adaptability has never been reported for any hydrolase‐like nanozyme and offers a distinct advantage over natural GHs, which are typically inactive at such low temperatures. Furthermore, when benchmarked against state‐of‐the‐art hydrolytic nanozymes reported to date, its performance was remarkably superior in terms of efficiency, especially under cold conditions (Table S3). To rule out the contribution of leached metal ions, the individual activities of Cu^+^, Cu^2+^, Mn^2+^, and Mn^3+^ were systematically examined, none of which showed measurable hydrolytic activity toward pNPG (Figure S3). Moreover, D‐CuMnO_x_ exhibited exceptional activity across a wide pH range and robust tolerance to thermal, high‐salt, and high‐organic solvent environments (Figure S4), highlighting its strong potential for practical applications.

Based on these findings, we investigated the hydrolytic activity of D‐CuMnO_x_ toward disaccharides containing glycosidic linkages. As an initial exploration, cellobiose, xylobiose, and maltose, each containing *β*‐1,4‐glycosidic bonds analogous to those in pNPG, were selected as models. As revealed by gas chromatography‐mass spectrometry (GC‐MS) results (Figure 2d‐f), the glycosidic bonds in these molecules were hydrolyzed, yielding glucose from cellobiose, xylose from xylobiose, and glucose from maltose. To further assess substrate versatility, the hydrolysis reaction was extended to maltose and sucrose, which contain *α*‐1,4‐ and *α*‐1,2‐glycosidic bonds, respectively. The results in Figure 2g,h demonstrate that, in both cases, the glycosidic bonds were effectively hydrolyzed, yielding the anticipated monosaccharide. These findings provide strong evidence for the broad‐spectrum hydrolytic capability of D‐CuMnO_x_, in contrast to previously reported mimics that generally exhibit limited substrate specificity [12]. This versatility suggests that the Lewis acid‐base sites in D‐CuMnO_x_ are not restricted to specific glycosidic geometries but rather facilitate the generalized activation of diverse linkages. Notably, D‐CuMnO_x_ maintained substantial hydrolytic efficiency across all tested substrates at 0°C compared to 37°C, highlighting its distinct cold‐adapted activity. To further quantify these transformations, the reaction mixtures were analyzed using the 3,5‐dinitrosalicylic acid (DNS) assay to measure the reducing sugar concentrations. In all cases, a significant increase in reducing sugar content was observed (Figure 2i), corroborating the effective hydrolysis of disaccharides into monosaccharides. Considering that the oxidase‐like activity of D‐CuMnO_x_ may lead to possible oxidation of the product, additional verification was performed using high‐performance anion‐exchange chromatography with pulsed amperometric detection (HPAEC‐PAD). Analysis of the reaction products from cellobiose treatment shows glucose as the only detectable product, with no aldonic acids observed (Figure S5). These results confirm that the D‐CuMnO_x_ does not further oxidize the hydrolysis products and therefore does not interference with the DNS‐based quantification. Collectively, these findings confirm that D‐CuMnO_x_ exhibits broad‐spectrum, cold‐adapted hydrolytic activity toward disaccharide decomposition.

### 2.3 GHs‐Like and Cold‐Adapted Catalytic Mechanism

**FIGURE 3:**
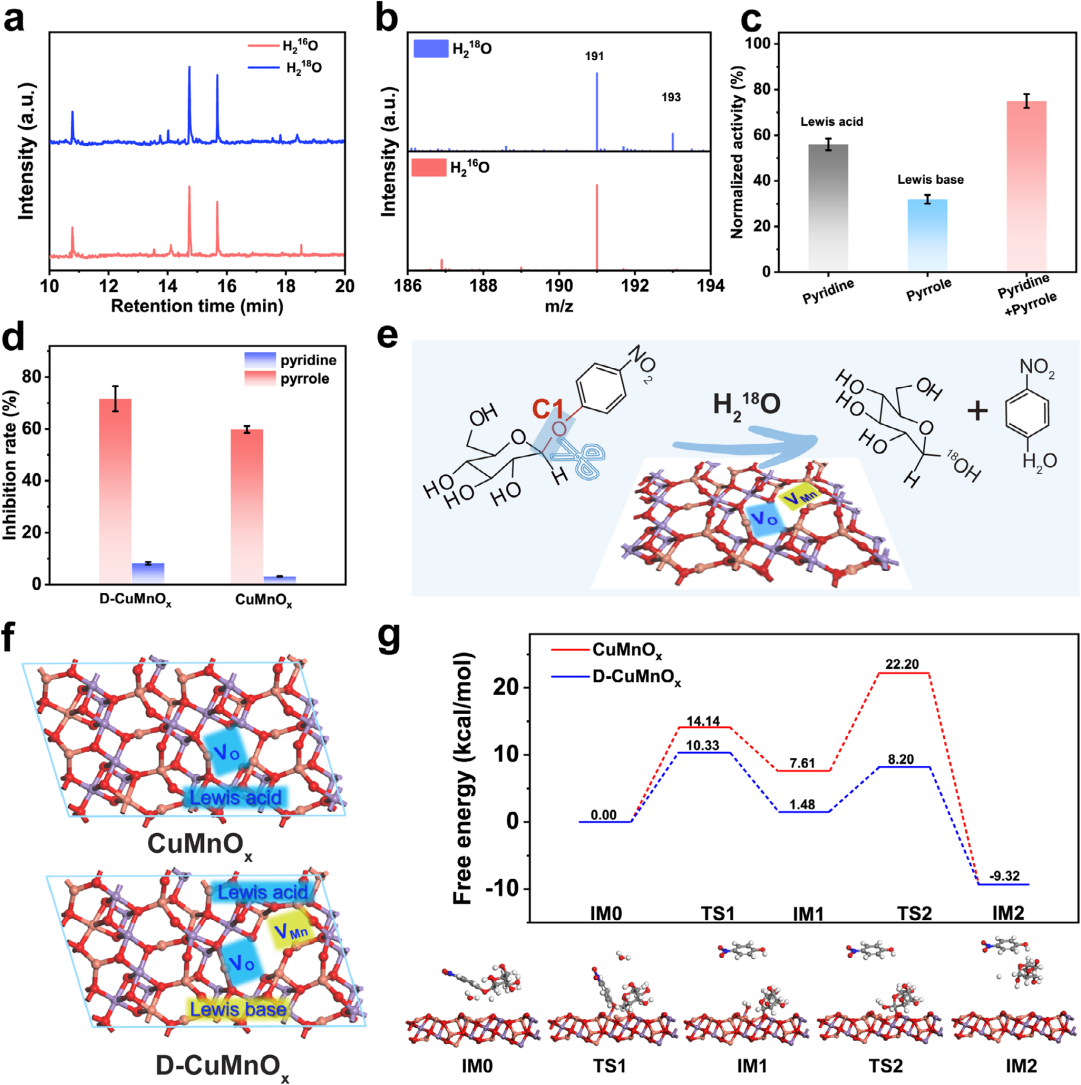
(a) GC‐MS spectra of the reaction system with H_2_^18^O labeling and (b) mass spectra of glucose. Quenching experiments targeting different active sites (c) at low temperatures (d). (e) Proposed hydrolytic mechanism of D‐CuMnO_x_. (f) Molecular models of CuMnO_x_ (top) and D‐CuMnO_x_ (bottom). (g) Energy profile of the hydrolysis reaction on the surface of the nanozyme and the corresponding indeterminate.

To further probe the reaction pathway and underlying mechanism, we employed isotopic labeling experiments using H_2_^18^O instead of H_2_^16^O to clarify the role of water in the pNPG hydrolysis reaction. As reflected in the GC‐MS spectra (Figure 3a), the released 4‐NP signals were identical in both H_2_^16^O and H_2_^18^O systems, indicating that the oxygen atom from water was not incorporated into this product. In contrast, the glucose derivatives exhibited distinct mass shifts, which were observed at m/z = 191 in the H_2_^16^O system and mainly at m/z = 193 in the H_2_^18^O system (Figure 3b). These results confirm that the reaction proceeds via a hydrolytic pathway, in which the glycosidic bond is cleaved at the acetal (or hemiacetal) carbon. Simultaneously, the water molecule (H_2_O) is cleaved, and the resulting hydroxyl group (‐OH) is incorporated into D‐glucose, in agreement with the mechanism of natural glycoside hydrolases (GHs) [31, 32, 33].

Next, we sought to identify the active sites responsible for hydrolytic activity, focusing on the contributions of Lewis acid and base sites and their cooperative action. To selectively block these sites, we performed quenching experiments with pyridine (a Lewis base) and pyrrole (a Lewis acid). Pyridine, by donating its lone pair of electrons, blocks the Lewis acid sites, preventing these sites from participating in the reaction. In contrast, pyrrole, as a Lewis acid, accepts electron pairs from Lewis base sites, thereby inhibiting their interaction with electron‐donating species and effectively blocking the basic sites [26]. Post‐inhibition CO_2_‐TPD and NH_3_‐TPD profiles confirmed the selectivity of the probes (Figure S6): pyrrole treatment nearly inhibited basic sites, while pyridine treatment strongly suppressed acidic sites, demonstrating effective and specific blocking of the respective surface sites. As shown in Figure 3c, inhibiting the Lewis acid sites reduced the activity by 46.8%, whereas blocking the basic sites led to a 29.2% decrease. Simultaneous inhibition of both sites caused a 76.4% loss of activity, emphasizing the crucial role of their cooperation in the efficient D‐CuMnO_x_ nanozyme. This is further supported by the minimal suppression observed in CuMnO_x_, due to its weak Lewis acidity and lack of basic sites. Notably, under low‐temperature conditions, D‐CuMnO_x_ retained high catalytic efficiency, whereas CuMnO_x_ showed a significant decrease in activity (Figure S7). Quenching experiments (Figure 3d) revealed that the inhibition of Lewis acid sites strongly suppressed the activity, whereas blocking basic sites caused only a minor reduction, indicating that Lewis acid sites are key drivers of low‐temperature catalysis. Additionally, the reported ZnN_4_ nanozyme, which maintains significant GHs‐like activity at 10°C, provides further evidence, although its cold‐adapted performance has not been fully verified [12]. Thus, a dual‐site catalytic mechanism can be proposed (Figure 3e): defective Mn in D‐CuMnO_x_ serves as a Lewis acid site, protonating the glycosidic oxygen during bond cleavage, whereas a neighboring O or oxygen vacancy acts as a general base to deprotonate the nucleophilic water molecule. The water then decomposes into OH and H, with electrons from pNPG transferring to the proton, thereby facilitating glycosidic bond cleavage and yielding glucose along with the leaving group, R‐OH. This cooperative action accelerates bond cleavage and ensures substrate selectivity by positioning the glycosidic bond in an optimal orientation for the reaction. In addition, weak signals of superoxide and its mixtures with alkyl radicals were detected in the reaction system (Figure S8), indicating that D‐CuMnO_x_, similar to the reported nanomimics, might also possess oxidative capability to attack glycosidic bonds [34, 35, 36]. The presence of weak mass spectrometry signals at m/z = 191 further supports this cooperative hydrolytic‐oxidative mechanism [37, 38].

Based on the above experimental findings, density functional theory (DFT) calculations were performed to gain theoretical insights. A D‐CuMnO_x_ model exposing the (311) crystal plane and incorporating both V_Mn_ and V_O_ was employed (Figure 3f; Table S4), and its performance was compared with that of CuMnO_x_ containing only V_O_ sites. As shown in Figure 3g, D‐CuMnO_x_ exhibited a relatively lower adsorption energy for pNPG, which is consistent with the reported concept that moderate adsorption strength favors hydrolytic activity. Furthermore, the calculated energy profiles revealed that D‐CuMnO_x_ required less energy to incorporate OH into glucose, which was identified as the rate‐determining step during hydrolysis. In addition, the overall energy barriers for hydrolysis were significantly reduced on D‐CuMnO_x_, indicating a thermodynamically more favorable pathway and providing theoretical validation that is consistent with the experimental observations.

### 2.4 Degradation of Polysaccharide

**FIGURE 4:**
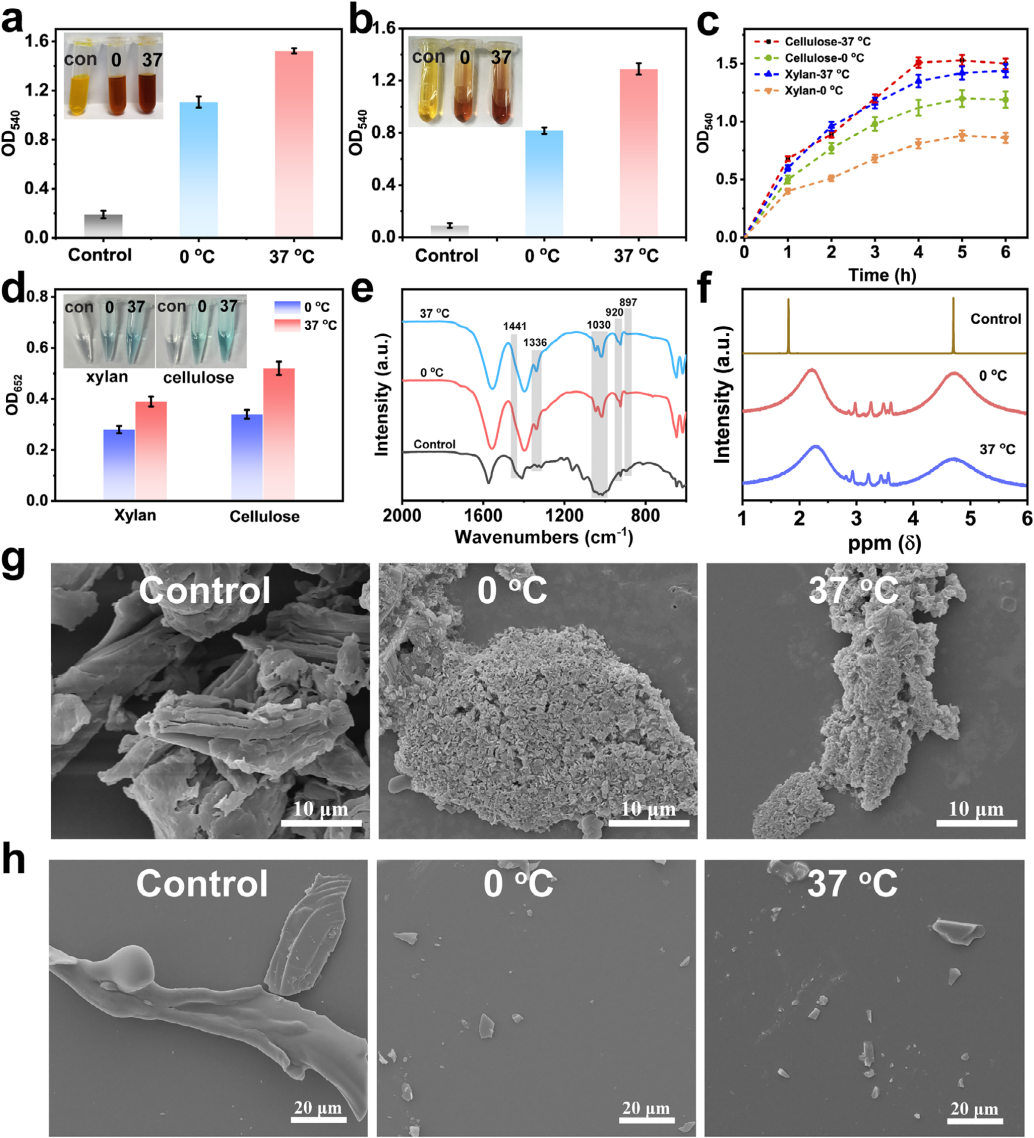
Degradation of polysaccharides by D‐CuMnO_x_. DNS results of cellulose system (a), xylan system (b), and (c) time‐dependent DNS results. (d) Glucose detection in both systems with corresponding observations (inset). (e) FTIR and (f) 1H NMR spectra of the cellulose system. (g, h) SEM images of the cellulose and xylan systems, respectively.

We further assessed the hydrolytic ability of D‐CuMnO_x_ toward polysaccharide polymers using cellulose and hemicellulose (xylan), which contain highly ordered *β*‐1,4‐glycosidic linkages, as model substrates. During treatment, the liquid supernatants were analyzed using the DNS assay. As shown in Figure 4a‐c, both cellulose and hemicellulose exhibited a noticeable color change from yellow to brownish‐red, reflecting the production of reducing sugars through the cleavage of glycosidic bonds. This observation was corroborated by glucose measurements that confirmed the presence of glucose in both systems (Figure 4d).

The structural changes that occurred during hydrolysis were systematically evaluated. The Fourier transform infrared (FTIR) results show a significant decrease in the 1030 cm^−1^ peak (Figure 4e), associated with C‐O‐C stretching of glycosidic linkages, alongside an increase at the peak of 1336 cm^−1^, corresponding to C‐H deformation vibrations, which suggest the cleavage of polysaccharide chains and formation of hydroxylated monosaccharides and oligosaccharides. There was also a reduction in crystallinity, as evidenced by a decrease in intensity at 1441 cm^−1^ (CH_2_ scissoring in crystalline regions) and an increase at 897 cm^−1^ (*β*‐glycosidic C‐H deformation in amorphous regions). The 1H NMR spectra (Figure 4f) of cellulose revealed characteristic peaks at approximately 2.0 and 4.5 ppm. Following treatment, multiple new peaks appeared in the 3.0–4.0 ppm region, corresponding to the chemical shifts of protons from newly generated oligosaccharides or monosaccharides. Morphological changes were also observed through SEM, where the long, strip‐like cellulose fibers were fragmented into smaller pieces, and the dense, compact structure of hemicellulose was broken into irregular nanoparticles (Figure 4g,h). This structural breakdown was accompanied by significant weight loss (Figure S9). Notably, these changes were largely preserved at 0°C, similar to those observed at 37°C, indicating that D‐CuMnO_x_ effectively depolymerized polysaccharides into monosaccharides and oligosaccharides, even at low temperatures.

### 2.5 Simultaneous degradation of lignin, cellulose and hemicellulose in corn stalk

Beyond hydrolytic activity, efficient lignocellulose degradation also depends on the cooperative contribution of oxidase‐like activity [39, 40], which prompted us to further investigate the oxidative capabilities of D‐CuMnO_x_. As shown in Figure S10, D‐CuMnO_x_ effectively catalyzed the oxidation of 2,4‐dichlorophenol (2,4‐DP) and 3,3’,5,5’‐tetramethylbenzidine (TMB), which are typical substrates for laccase‐ and oxidase‐like reactions, respectively, confirming its broad‐spectrum oxidative activity in this regard. In biological systems, oxidative enzymes, including laccases, mediate lignin degradation by initiating electron transfer and radical chain reactions, preferentially targeting the *β*‐O‐4 linkage. This linkage accounts for more than 50% of the interunit bonds in lignin and has a relatively low bond dissociation energy, making it a primary cleavage site. Cleavage of the *β*‐O‐4 bond induces backbone depolymerization, which in turn exposes and facilitates the subsequent cleavage of other linkages, such as *β*‐5 and *β*‐*β*, promoting further lignin depolymerization. In light of this, the oxidative potential of D‐CuMnO_x_ was assessed using guaiacylglycerol‐β‐guaiacyl ether (GGE) as a model compound. Combined HPLC analysis (Figure S11a) and UV–vis spectroscopy (Figure S11b) revealed that D‐CuMnO_x_ efficiently cleaved the *β*‐O‐4 bond, yielding guaiacol as the primary product, highlighting its potential for the oxidative degradation of lignin.

**FIGURE 5:**
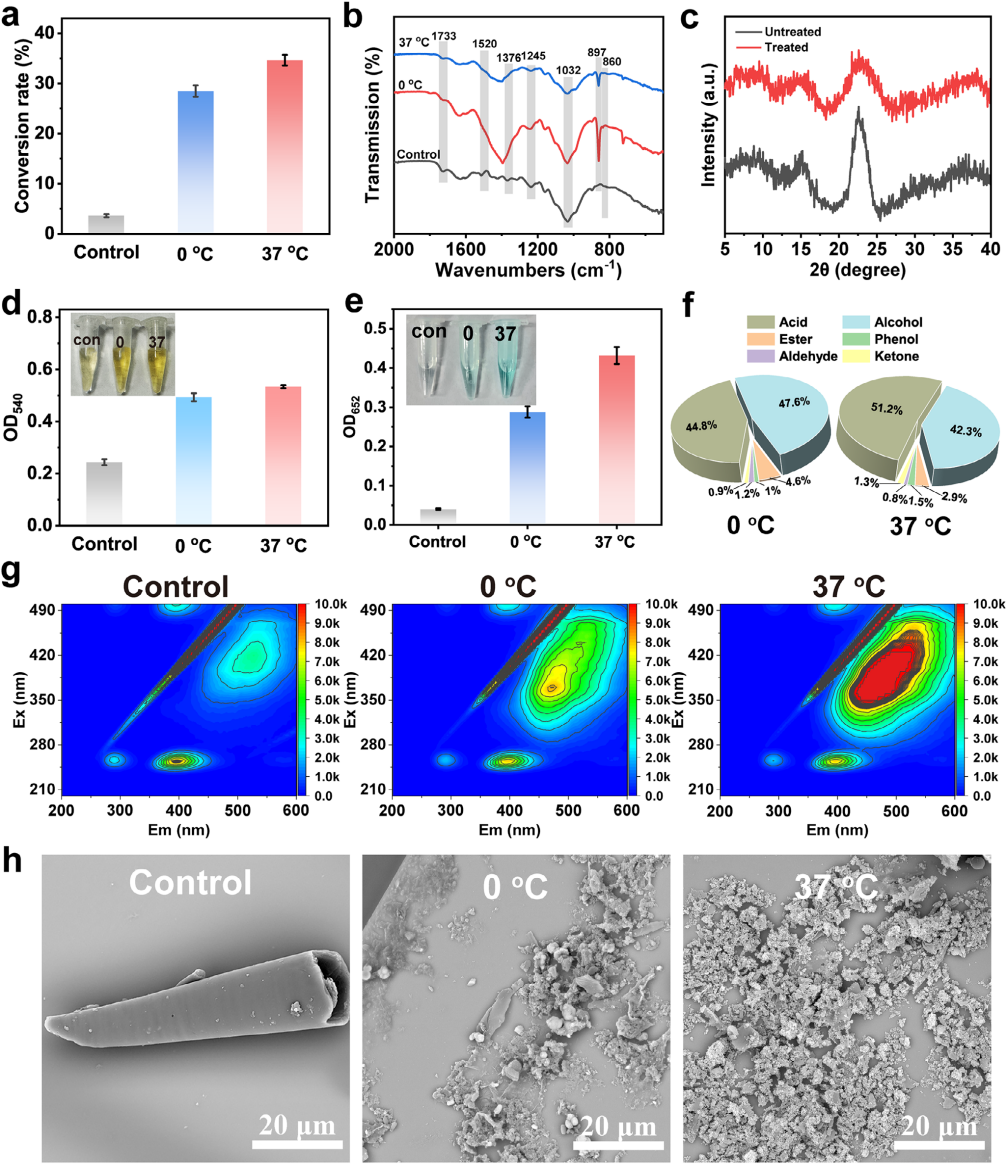
Degradation of corn stalks by D‐CuMnO_x_. (a) Conversion rate determined by weight loss. (b) FTIR and (c) XRD spectra of the treated samples. (d) DNS assay, (e) glucose kit assay and (f) production distribution from GC‐MS assay of reaction system. (g) 3D EEM spectra of the reaction system and (h) SEM images of the samples.

Leveraging the advanced hydrolytic and oxidative properties of D‐CuMnO_x_, we evaluated its potential for depolymerization of three major components within one system. Raw corn stalk, a representative natural lignocellulose, was selected as the substrate, and reactions were performed at 0°C and 37°C for comparison. After treatment, significant weight loss was observed, with a conversion rate of 35.7% at 37°C and 28.5% at 0°C (Figure 5a). FTIR analysis (Figure 5b) revealed significant changes in structure, with reductions in peaks at 1520 cm^−1^ and 1254 cm^−1^, corresponding to benzene rings and carbonyl groups in lignin, and at 1730 cm^−1^, related to hemicellulose acetyl esters, indicating the degradation of lignin and hemicellulose. Cellulose also underwent hydrolysis, as evidenced by a decrease in the peaks at 1367 cm^−1^ and 1062 cm^−1^. The appearance of a peak at 860 cm^−1^, associated with the amorphous region, indicates disruption of the crystalline structure. XRD analysis, which is widely used to evaluate the crystallinity of lignocellulosic biomass by probing its supramolecular structure [41, 42], further confirmed these structure changes. As shown in Figure 5c, the characteristic diffraction peaks became broader and less intense after treatment, indicating a reduced crystalline order and a concomitant increase in amorphous domains. The degradation of cellulose and hemicellulose was quantified using the DNS method (Figure 5d), which presented a dark brown color in the treated samples, indicating the generation of reducing sugars. The degradation behavior was also confirmed from the glucose production within these systems (Figure 5e).

GC‐MS analysis (Table S5) was performed to investigate the main chemical components of the products, which were categorized into six groups based on their functional groups: alkanes, alcohols, acids, ethers, esters, and phenolic compounds (Figure 5f). Among these, acids and alcohols are the dominant components, suggesting a transformation process that involves both hydrolysis and oxidation. Furthermore, various aromatic and long‐chain aliphatic compounds were detected, indicating C─C/C─O bond cleavage and ring‐opening reactions during degradation. Notably, several value‐added chemicals, including dibutyl phthalate and 3, 4‐dihydroxymandelic acid, were identified in the system, highlighting the potential for biotransformation applications. 3D excitation‐emission matrix (3D EEM) fluorescence spectroscopy was used to analyze the dissolved organic matter in the treated samples. As shown in Figure 5g, the humic‐like region (*E_x_/E_m_*: 300–450/400–600 nm) exhibited a red shift and signal diffusion, indicating lignin‐derived aromatic polymers with extended electron delocalization. A decrease in fluorescence intensity in the fulvic acid‐like region (*E_x_/E*_m_: 240–260/380–440 nm) suggested the oxidative breakdown of low‐molecular‐weight components. These changes were reflected in the visible morphology (SEM images, Figure 5h), from intact, smooth rod‐like structures to fragmented pieces. These trends were observed at both 0°C and 37°C, with more pronounced changes at 37°C. To track the transformation of biomass components, the total organic carbon (TOC) content in the raw corn stalk, residual solids, and liquid products was quantified. As shown in Table S6, the calculated carbon efficiency reached 30.03% at 37°C and 24.8% at 4°C, reflecting efficient redistribution of carbon into soluble products. Complementary mass balance calculations achieved near closure (discrepancies typically <10%) at both temperatures. The minor deviations might be attributed to sample handling losses during the determination process or trace gaseous emissions under the mild reaction conditions. Furthermore, mass analysis of the three major components before and after treatment (Table S7) revealed substantial reductions across all fractions, with lignin showing the highest degradation rate, followed by hemicellulose and cellulose. These quantitative changes, combined with GC‐MS product profiling, FTIR, SEM, and 3D‐EEM evidence, collectively confirm the exceptional ability of D‐CuMnO_x_ to efficiently degrade lignin, hemicellulose, and cellulose in corn stalks across a wide temperature range.

Based on the obtained results, we propose a potential oxidative‐hydrolytic mechanism that mimics the action of natural ligninolytic enzyme consortia, in which radical‐mediated processes initiate bond cleavage in lignin and thereby facilitate subsequent access to polysaccharides. Guided by the efficient cleavage of the *β*‐O‐4 model compound GGE, the corresponding radical detection results, and the highest degradation rate observed for lignin among the three major components, the nanozyme appears to preferentially target the most labile *β*‐O‐4 linkages as the primary attack point. This initiates a chain‐propagation process that further oxidizes recalcitrant lignin motifs, leading to pronounced structural disruption. The initial breakdown of lignin exposes cellulose and hemicellulose, enabling their concurrent hydrolysis into soluble sugars and oligomers. Overall, this cooperative dual functionality under mild conditions enables the simultaneous degradation of all three major components, thereby offering strong potential for full‐component utilization and value‐added transformation of lignocellulosic biomass.

## 3 Conclusions

In summary, we developed a cold‐adapted multifunctional nanozyme of D‐CuMnO_x_ by integrating Cu doping with amorphous engineering, which simultaneously introduced Mn and oxygen vacancies. These defect‐induced Lewis acid‐base pairs act as cooperative catalytic centers, enabling D‐CuMnO_x_ to exhibit both hydrolytic and oxidative activities, together with remarkable cold adaptability. Mechanistic studies with isotopic labeling confirmed that Lewis pairs facilitate proton transfer and water activation for glycosidic bond cleavage. Supported by DFT calculations, the lowered energy barriers and optimized adsorption states rationalized the superior activity. Consequently, D‐CuMnO_x_ catalyzes the broad‐spectrum hydrolysis of disaccharides and polysaccharides even at 0°C, and further drives the simultaneous depolymerization of cellulose, hemicellulose, and lignin in raw corn stalks. Furthermore, the robust cold adaptability of D‐CuMnO_x_ highlights its potential for sustainable biomass valorization, whereas conventional biocatalysts fail. This study not only establishes a conceptual framework for designing multi‐nanozymes with integrated cold‐adapted functions, but also opens avenues for complete lignocellulose utilization and green biotransformation for value‐added chemicals.

## 4 Materials and Methods

### 4.1 Materials

Manganese(II) sulfate (MnSO_4_), Copper(II) sulfate (CuSO_4_), xylobiose, cellobiose, maltose, lactose, sucrose, cellulose, 3,3',5,5'‐Tetramethylbenzidine (TMB), 2,4‐dichlorophenol, guaiacylglycerol‐*β*‐guaiacyl, pyridine, pyrrole and regular organic solvent were purchased from Shanghai Macklin Biochemical. Fructose, galactose, glucose and xylose were purchased from Sinopharm Chemical Reagent Ltd. Xylan were purchased from Shanghai Yuanye Bio‐Technology.

### 4.2 Characterizations

The morphology of materials were observed with the transmission electron microscope (FEI Talos F200X TEM) and scanning electron microscopy (SEM, VEGA 3 SBH). X‐ray diffraction (XRD) patterns were collected using a D8 DISCOVER A25 diffractometer over the 2θ range of 10–80° with Cu K*α* radiation. The electronic structure and surface chemical states of the MOFs were analyzed by X‐ray photoelectron spectroscopy (XPS, Thermo ESCALAB 250) equipped with a monochromatic Al K*α* radiation source. X‐ray absorption spectroscopy, including X‐ray absorption near‐edge structure (XANES) and extended X‐ray absorption fine structure (EXAFS) measurements at the Mn K‐edge, were carried out at the BL08U1A and BL14W1 beamlines of the Shanghai Synchrotron Radiation Facility (SSRF). Fourier transform infrared (FTIR) spectra were recorded using a Nicolet iS50 Spectrometer (Thermo Fisher, USA) in the range of 4000–600 cm^−1^ with a resolution of 2 cm^−1^. UV–vis absorption spectra were obtained using a microvolume spectrophotometer (Nanodrop OneC, Thermo Fisher Scientific, USA). Inductively coupled plasma‐atomic emission analysis (ICP‐AES) measurements were performed on a Optima 8300 ICP Spectrometer (Perkin Elmer). Electron spin resonance (ESR) measurements were performed using 5,5‐dimethyl‐1‐pyrroline N‐oxide (DMPO) as the spin‐trapping agent. 20 µg of D‐CuMnO_x_, 100 µL of pNPG solution (30 mM), and 760 µL of acetate buffer (pH 4.0) were sequentially added into a 1.5 mL centrifuge tube, followed by the addition of 40 µL of DMPO under continuous stirring. The ESR spectra were recorded on a Bruker EMX PLUS spectrometer.

CO_2_‐TPD and NH_3_‐TPD analyses were conducted on a Micromeritics AutoChem II 2920 instrument. About 50–100 mg of sample was pretreated in He flow at 200°C for 1 h to remove adsorbed species. After cooling to 50°C (for CO_2_) or 100°C (for NH_3_), the sample was saturated with 10 vol% CO_2_/He or 10 vol% NH_3_/He, followed by He purging to eliminate physiosorbed molecules. Desorption was performed by heating to 800°C at 10°C·min^−1^ under He, and the signals were recorded with a TCD detector.

### 4.3 Synthesis of D‐CuMnO_x_

D‐CuMnO_x_ was synthesized following reported procedures by integrating amorphous engineering with Cu doping [1, 2]. Briefly, 0.9 mmol of CuSO_4_ was introduced into a mixed solution containing 1.35 mmol of MnSO_4_ and 33.75 mmol of triethanolamine (TEA). After stirring for 5 min, ammonia solution was added, and the mixture was continuously stirred at 50°C for 12 h. The resulting precipitate was collected by centrifugation, thoroughly washed with ethanol and deionized water, and subsequently dried overnight at 70°C. The obtained material was denoted as CuMnO_x_. To generate Mn vacancies, CuMnO_x_ was further treated with nitric acid according to a previously reported method, yielding the final D‐CuMnO_x_.

### 4.4 Nanozymatic Activity Assay

#### 4.4.1 Glucosidase‐Like Activity Assay

The hydrolase‐like activity of the sample was evaluated using p‐nitrophenyl‐*β*‐D‐glucopyranoside (pNPG) as the substrate. In a typical assay, nanozyme suspension (5 mg·mL^−1^, 50 µL) and pNPG solution (50 mM, 20 µL) were added into sodium acetate–acetic acid buffer (pH 4.0, 930 µL). The reaction was carried out at 37°C under continuous stirring. After completion, 27 µL of 1 mM NaOH solution was introduced to quench the reaction. The mixture was then filtered and centrifuged, and the absorbance of the supernatant was recorded at 405 nm using a UV–vis spectrophotometer. To investigate cold‐adapted performance, the activity assay was also performed at different temperatures (50, 37, 0, and −10°C).

#### 4.4.2 Laccase‐Like Activity

The laccase‐like activity of the samples was examined using 2,4‐dichlorophenol (2,4‐DCP) as the substrate. In a typical assay, 100 µL of 2,4‐DCP solution (10 mM) and 100 µL of 4‐aminoantipyrine (4‐AP, 10 mM) were mixed with an appropriate amount of nanozyme suspension in MES buffer (0.1 M, pH 6.8). The total reaction volume was adjusted to 1 mL. The reaction was carried out at 37°C under gentle stirring. The catalytic oxidation of 2,4‐DCP coupled with 4‐AAP produced a red‐colored product, and the absorbance was monitored at 510 nm using a UV–vis spectrophotometer. Control experiments in the absence of nanozyme were performed under identical conditions.

#### 4.4.3 Oxidase‐Like Activity

The oxidase‐like activity of the nanozyme was assessed using 3,3′,5,5′‐tetramethylbenzidine (TMB) as the chromogenic substrate. Upon catalytic oxidation, TMB yielded a characteristic blue product exhibiting absorption peak at 370 and 652 nm. For a typical assay, 10 µL of TMB solution (25 mM) and nanozyme with varying concentrations were added sequentially to sodium acetate‐acetic acid buffer (0.2 M, pH 3.6), and the total reaction volume was adjusted to 1 mL. The absorbance of the system at 652 nm was continuously monitored in kinetic mode using a UV–vis spectrophotometer.

### 4.5 Degradation of Disaccharide and Polysaccharide Substrates

In a typical assay, 100 µL of nanozyme suspension (2 mg·mL^−1^), 100 µL of xylobiose solution (30 mM), and 800 µL of acetate buffer (pH 4.0) were sequentially added into an Eppendorf tube. After the reaction, the mixture was centrifuged at 14 000 rpm for 10 min to collect the supernatant, which was subsequently filtered through a 0.22 µm membrane filter (Amber Laboratory Technology Co., Ltd., China). The filtrate was freeze‐dried to completely remove the solvent, redissolved in 200 µL of pyridine, and derivatized with 100 µL of BSTFA at 100°C for 1 h. The derivatized solution was dried over anhydrous sodium sulfate to eliminate residual moisture before gas chromatography‐mass spectrometry (GC‐MS) analysis.

GC‐MS analysis was performed on an Agilent 7000D gas chromatograph coupled with an Agilent 5975 mass selective detector, equipped with an Agilent HP‐5 ms capillary column (30 m × 250 µm × 0.25 µm). The chromatographic program was set as follows: the initial column temperature was raised from room temperature to 80°C and held for 3 min, then ramped to 280°C at 10°C·min^−1^ and maintained for 5 min, followed by an increase to 300°C at 20°C·min^−1^ with a final hold of 6 min. High‐purity helium was used as the carrier gas at a constant flow rate of 1 mL·min^−1^. The injector temperature was maintained at 280°C, and the injection volume was 1 µL. Degradation assays toward other substrates (cellobiose, maltose, lactose, and sucrose) were conducted under identical conditions. High‐performance anion‐exchange chromatography with pulsed amperometric detection (HPAEC‐PAD) were conducted on the treated cellobiose for further product analysis. The measurements were performed using a CarboPac PA20 column at 30°C with 10 mM NaOH as the eluent at a flow rate of 0.5 mL min^−1^. Glucose, cellobiose, and aldonic acid were used as reference standards for product identification. Product species were assigned by comparing their retention times with those of the standards.

Typically, 200 mg of xylan was dispersed in 20 mL of ultrapure water containing 20 mg of nanozyme. For cellulose degradation, 500 mg of cellulose was treated with 100 mg of nanozyme in 20 mL of water. Both suspensions were incubated with shaking at 0°C or 37°C. After the reaction, the mixtures were centrifuged at 10 000 rpm for 10 min, and the supernatants were filtered through a 0.22 µm membrane. To determine the soluble reducing sugar content, 1 mL of the supernatant was mixed with 1 mL of 3,5‐dinitrosalicylic acid (DNS) reagent in a 4 mL tube for subsequent analysis. The mixture was heated in a boiling water bath for 5 min, and the absorbance was subsequently measured at 540 nm. The glucose concentration in the reaction system was further determined using a coupled enzymatic assay with glucose oxidase (GOx) and horseradish peroxidase (HRP). In a typical experiment, 100 µL of the reaction supernatant mixed with 900 µL of phosphate buffer (0.1 M, pH 7.0) containing 1 U·mL^−1^ GOx. After complete reaction, 0.5 U·mL^−1^ HRP and 1 mM 3,3′,5,5′‐tetramethylbenzidine (TMB) were added and incubated at 37°C for 15 min. The absorbance of the resulting solution was measured at 652 nm using a UV–vis spectrophotometer. The morphology and microstructure of the polysaccharides, both before and after treatment, were characterized using SEM, FTIR, and nuclear magnetic resonance (NMR) spectroscopy.

### 4.6 DFT Calculations

The density functional theory (DFT) calculations were performed using a Castep module of Material Studio 2020 [3–5]. The generalized gradient approximation (GGA) method with Perdew‐Burke‐Ernzerhof (PBE) function was employed to describe the interactions between the valence electrons and the ionic core. The energy cut‐off for the plane‐wave basis set was 400 eV. The threshold values of the convergence criteria were specified as follows: 0.002 Å for maximum displacement, 0.05 eV Å^−1^ for the maximum force, 0.1 GPa for the maximum stress, 10^−5^ eV/atom for energy, and 2.0 × 10^−6^ eV/atom for self‐consistent field tolerance. The (311) surface of Mn3Cu3O8 (JCPDS NO. 71–3511) was cleaved. 2 layers of cell thickness are chosen to approximate the bulk properties. Based on Mn3Cu3O8(311), the CuMnO_x_ (311) was built to possess one O vacancy and D‐CuMnO_x_ (311) had one O vacancy and one Mn vacancy. The Brillouin zone integration is performed using a 2×2×1 k‐mesh. 15 Å vacuum space were implemented into the model to eliminate undesirable interactions between bottom side of the slab and the molecules in the vacuum space. Energy barriers were examined by linear and quadratic synchronous transit methods in combination with the conjugated gradient refinement. Spin polarizations are also considered in all calculations. The free energies (G) of different intermediates are defined as ΔG = *E*_i_‐*E*_reactant_ (*E*_i_ the energy of intermediates and *E*_reactant_ the total energy of reactants).

### 4.7 Degradation of GGE

In a typical experiment, 100 µL of D‐CuMnO_x_ solutions (2 mg·mL^−^^1^), 100 µL of GGE solution (30 mM), and 800 µL of acetate buffer (pH 4.0) were sequentially added into a tube (1.5 mL). After reaction, the mixtures were collected and filtered with 0.22 µm syringe filters, followed by analysis using UV–vis spectroscopy and high‐performance liquid chromatography (HPLC) at 280 nm. The HPLC test conditions were set as follows:

System: Waters Alliance HPLC system
Column: InertSustain C18
Eluent: A) CH_3_OH; B) Imidazole buffer; A/B = 15/85, v/v
Flow Rate: 0.85 mL/min
Col. Temp: 40°C
Injection Vol: 80 µL
Sample: Standard

Imidazole buffer: 68.08 g of imidazole, 0.37 g of disodium ethylenediaminetetraacetate and 10.72 g of magnesium acetate were dissolved in 800 mL H_2_O. The pH was adjusted to 7.2 with acetic acid and the volume of solution was fixed to be 1000 mL with ddH_2_O.

### 4.8 Degradation of Corn Stalk

In a typical experiment, 50 mg of straw lignocellulose was dispersed in 5 mL of ultrapure water, and 10 mg of D‐CuMnO_x_ was added. The mixtures were incubated with shaking at 0°C and 37°C. After the reaction, the supernatant was collected by centrifugation at 10,000 rpm for 10 min and subsequently filtered through a 0.22 µm membrane. To evaluate the degradation of straw lignocellulose, the precipitate was collected and dried at 105°C for 12 h. The morphology and chemical structure of the treated samples were characterized by FTIR, X‐ray diffraction (XRD), and scanning electron microscopy (SEM). The degradation rate of straw was calculated using the following formula:

$$\mathrm{Straw}\;\mathrm{degradation}\;\mathrm{rate}=\frac{m_{1}-m_{2}}{m}\times 100\%$$

where m_1_ represents the initial mass of the mixture, m_2_ is the mass of residual precipitate, m is the initial mass of the corn stalk.

The collected supernatants were analyzed by 3D excitation‐emission matrix (3D EEM) fluorescence spectroscopy to characterize the dissolved organic matter. To monitor changes in the soluble reducing sugar content, the supernatants were further assessed using the DNS assay, following the procedure described above in the Degradation of Polysaccharides section.

Raw corn stalk (100 mg, dry basis) was treated with D‐CuMnO_x_ under identical conditions at 37°C and 4°C. After the reaction, the mixture was separated into a solid residue and a liquid phase by centrifugation or filtration. Both fractions were dried to constant weight, and their masses were recorded. Mass efficiency was defined as the percentage of the total mass transferred from the raw corn stalk to the combined solid residue and liquid product. Notably, the liquid phase was dried at 105°C until a stable mass was obtained prior to weighing. The initial carbon content of the raw corn stalk (W_1_, mg) was determined using an elemental analyzer via high‐temperature combustion and CO_2_ detection. The total organic carbon (TOC) in the liquid phase (W_2_, mg) was quantified using a TOC analyzer operating in non‐purgeable organic carbon mode: the samples were first acidified to remove inorganic carbon, and the remaining organic carbon was oxidized to CO_2_ at 680–950°C over a platinum catalyst, followed by non‐dispersive infrared detection. Owing to the mild reaction conditions, gas‐phase carbon losses were considered negligible. Carbon efficiency was defined as the percentage of total carbon transferred from the raw corn stalk to the liquid‐phase products after treatment and was calculated as (W_2_/W_1_)×100%. All measurements were performed in triplicate, and average values are reported.

The contents of hemicellulose, cellulose, lignin, and ash in the raw corn stalk and post‐degradation solid residues were determined using a modified acid hydrolysis procedure. Samples were ground and passed through a 40‐mesh sieve, then dried at 105°C to constant weight (recorded as W_0_). The dried sample was treated with 1 M HCl and heated in an 80°C water bath for 1 h with intermittent stirring. The mixture was then filtered, and the residue was washed with hot distilled water until neutral and dried at 105°C to constant weight (W_1_). Hemicellulose content was calculated as (W_0_‐W_1_)/W_0_×100%. The residue (W_1_) was subsequently treated with 72% sulfuric acid at 20°C for 2 h with periodic stirring, diluted to ≤3% sulfuric acid, and boiled for 30 min. After filtration and washing until neutral, the residue was dried at 105°C to constant weight (W_2_). Cellulose content was calculated as (W_1_‐W_2_)/W_0_ × 100%. The final residue (W_2_) was incinerated in a muffle furnace at 550°C for 2 h, cooled, and weighed as ash (W_3_). Lignin content was calculated as (W_2_‐W_3_)/W_0_ × 100%. All measurements were performed in triplicate, and average values are reported.

### 4.9 Statistical Analysis

For all quantitative analyses, data are expressed as the mean ± SD, with each value calculated from five independent experimental trials (n = 3). The SD is visualized via error bars in the figures, which illustrate the degree of variation between replicate experiments.

## Conflicts of Interest

The authors declare no conflicts of interest.

## Supporting information




**Supporting File**: advs74418‐sup‐0001‐SuppMat.docx.

## Data Availability

The data that support the findings of this study are available from the corresponding author upon reasonable request.
