# Supplementary material for: Lewis Pair‐Engineered CuMnOx as Cold‐Adapted Multinanozyme for Cooperative Hydrolytic and Oxidative Degradation of Raw Corn Stalk
Source: Adv Sci (Weinh). 2026 Feb 15;13(23):e19235. doi: 10.1002/advs.202519235 (PMC13104106; doi:10.1002/advs.202519235)
Supplement: Supplementary file 1 — Supporting File: advs74418‐sup‐0001‐SuppMat.docx. [file ADVS-13-e19235-s001.docx]

**Supporting Information**

**Lewis Pair Engineered CuMnO_x_ as Cold-Adapted Multinanozyme for Cooperative Hydrolytic and Oxidative Degradation of Raw Corn Stalk**

*Huile Liu ^a,b,†^, Ziyi Di ^a,b,†^, Qing Tian ^a,b^, Yue Zhou ^a^, Rong Yang ^a,b^, Zhiyi Bai ^a,b^, Haoyu Wang ^a^, Yao Chen ^a,b,*^* and *Lianbing Zhang ^a,b,*^*

Email: chenyao@nwpu.edu.cn and lbzhang@nwpu.edu.cn

1. School of Life Sciences and Technology, Northwestern Polytechnical University, Xi’an 710072, China
2. Nanozyme Laboratory in Zhongyuan, Henan Academy of Innovations in Medical Science, Zhengzhou 451163, China

**
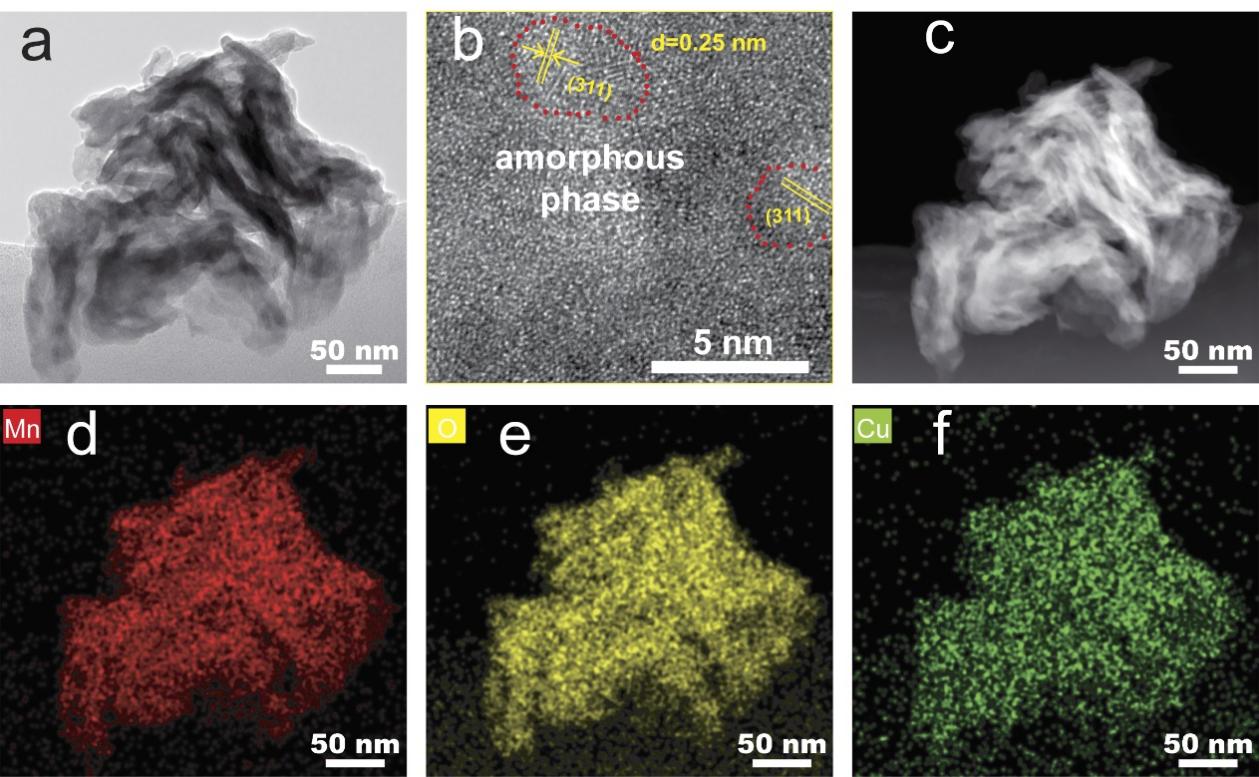
**

Fig S1. (a) TEM, (b, c) HRTEM and (d) HAADF images of CuMnO_x_ with corresponding EDS mapping (Mn, Cu and O elements).


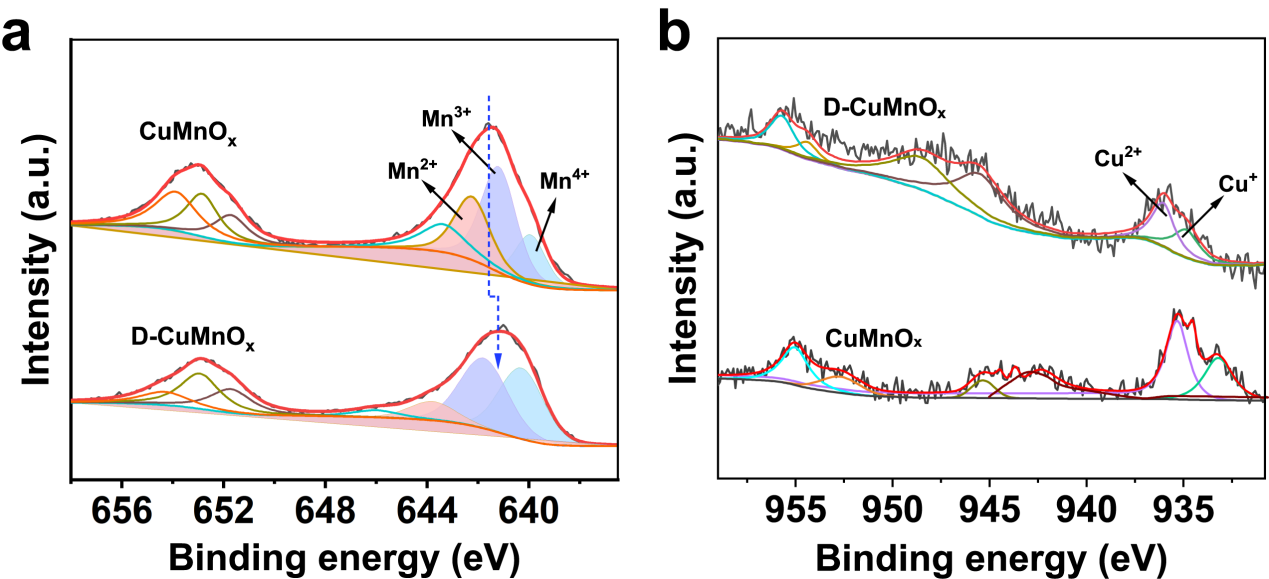


Fig S2. Mn2p (a) and Cu 2p (b) XPS spectra of DCuMnO_x_ and CuMnO_x_.


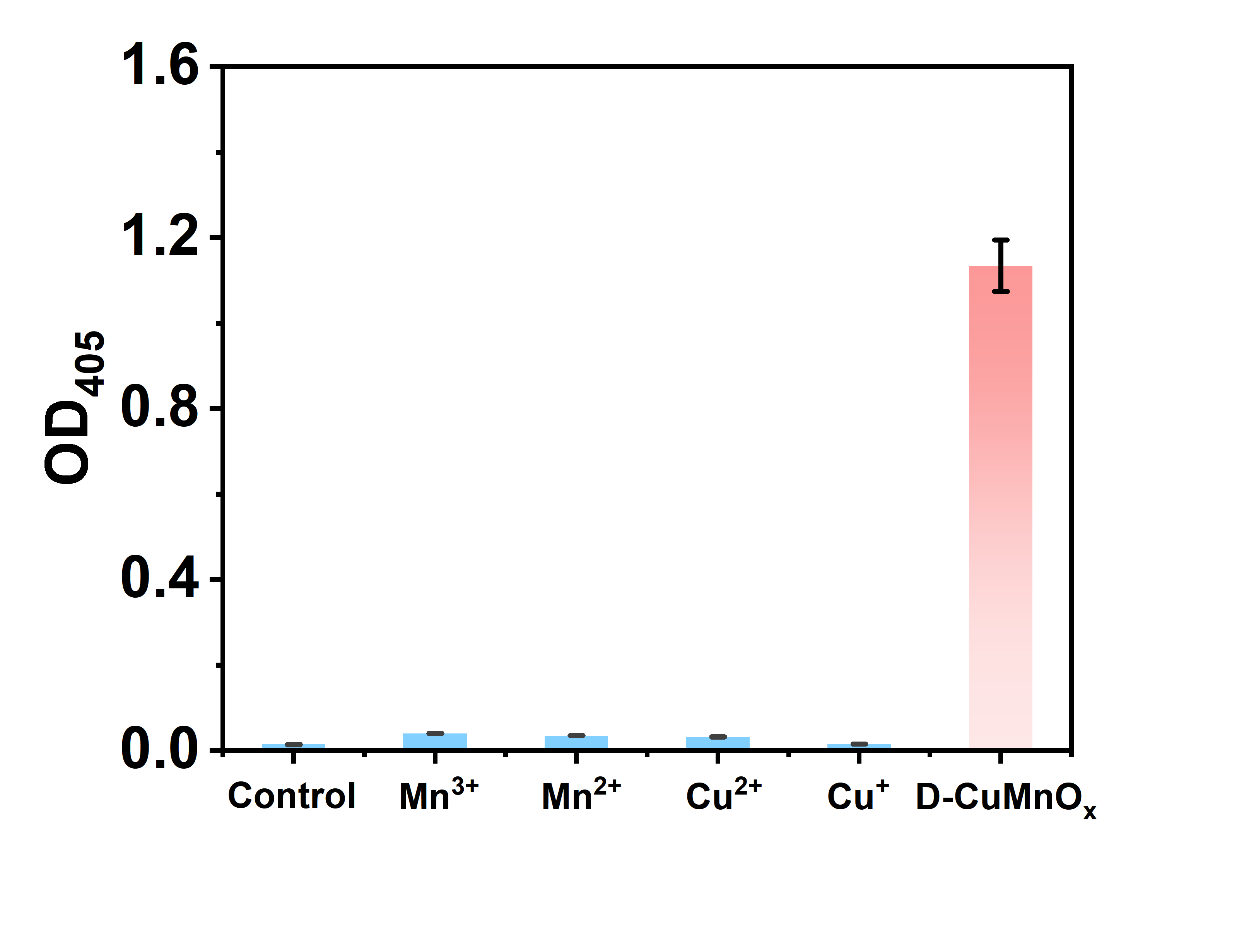


Fig S3. UV-vis spectra for pNPG hydrolysis catalyzed by different metal ions.


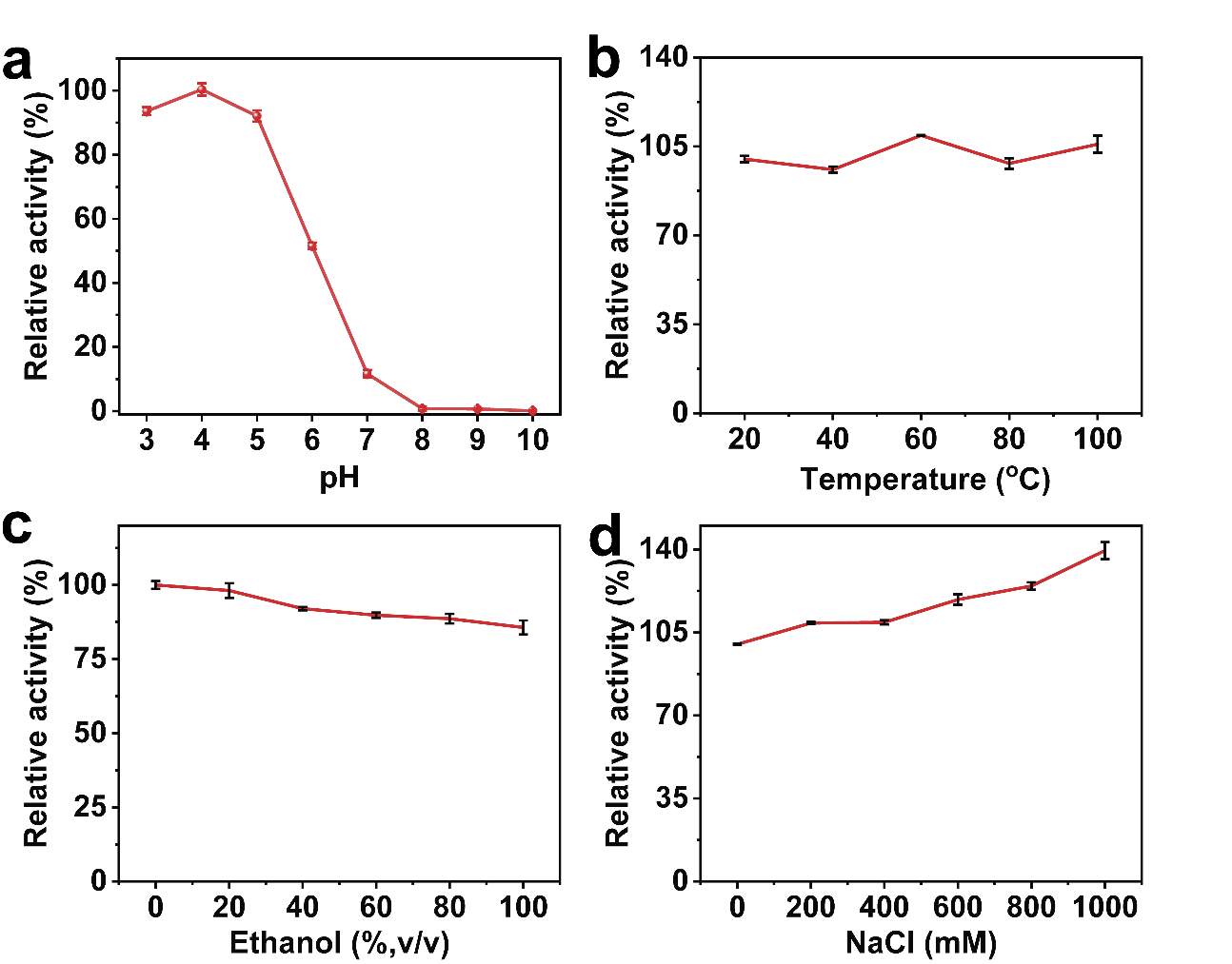


Fig S4. Catalytic activity assay under different pH (a), after cultivation at different temperature for 3 h (b), in system with varied concentration (c) and NaCl (d).


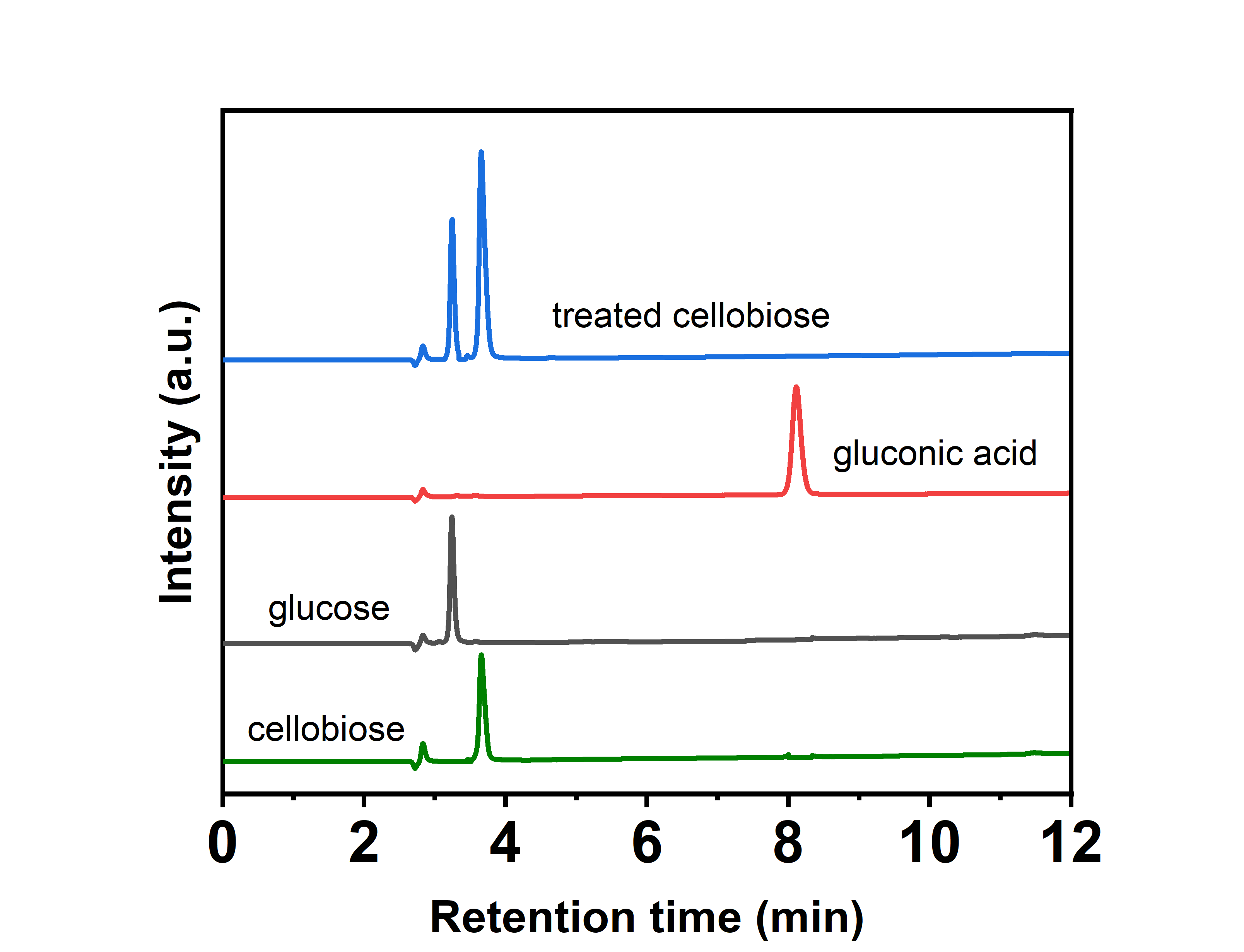


Fig S5. The HPAEC-PAD analysis result of treated cellobiose.


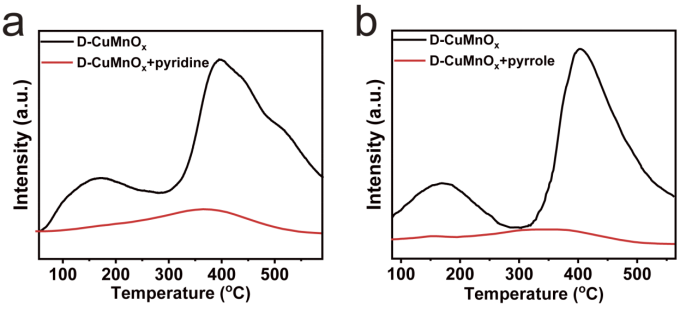


Fig S6. The NH_3_-TPD profile of D-CuMnO_x_ after pyridine adsorption (a) and CO_2_-TPD profile of D-CuMnO_x_ after pyrrole adsorption (b).


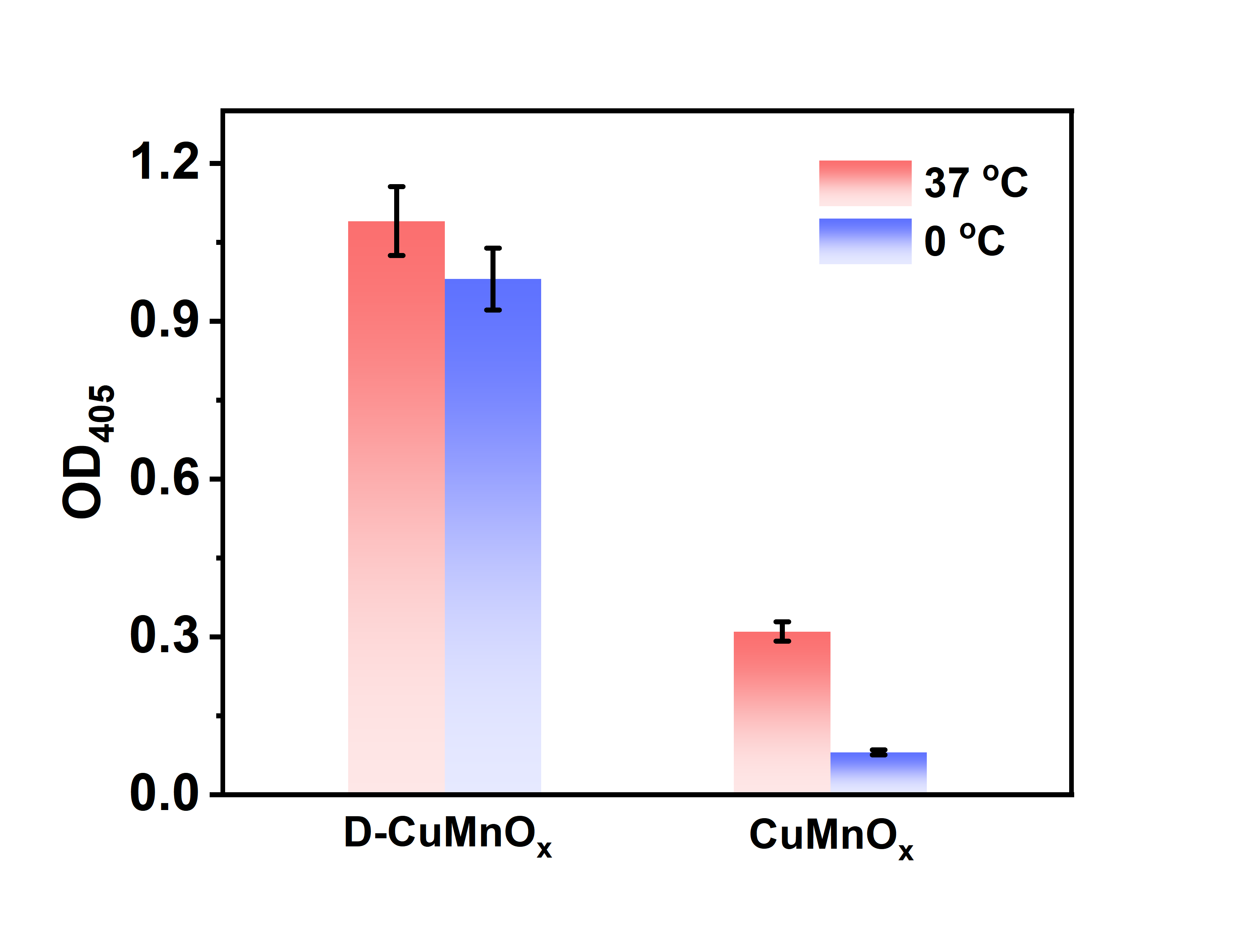


Fig S7. Cold-adapted activity comparison under different temperatures.


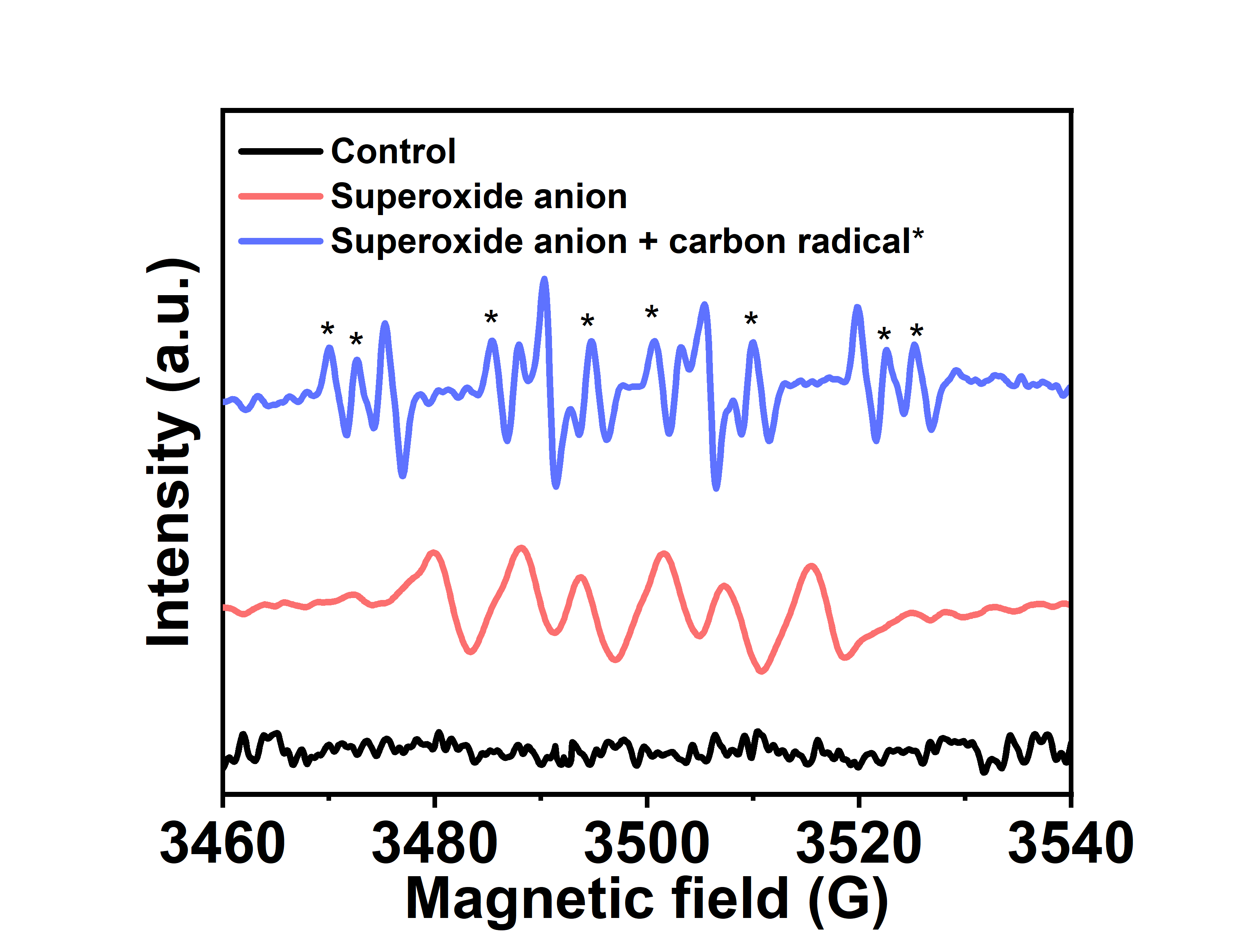


Fig S8. EPR spectra of the reaction system using DMPO as a spin-trapping agent. Signals are observed in both the red and blue line.


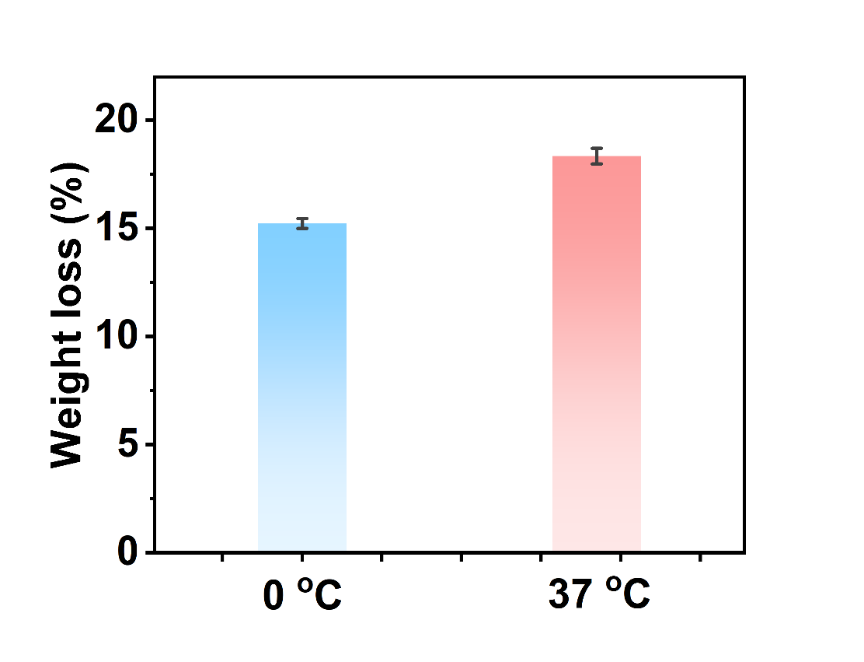


Fig S9. Weight loss of cellulose after treatment at different temperatures.


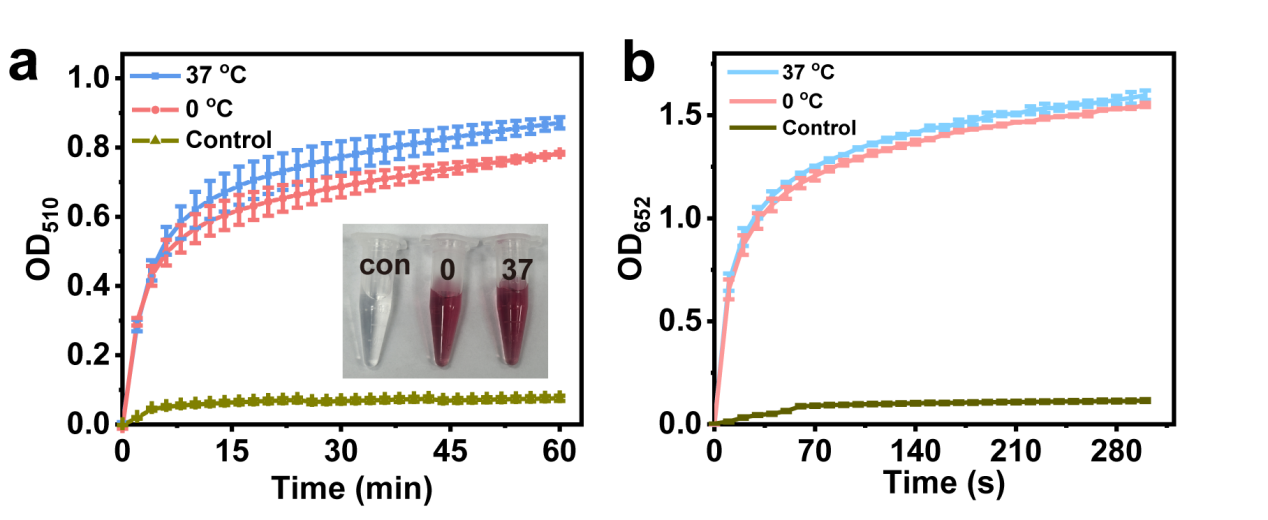


Fig S10. UV-vis adsorption value of laccase (a) and oxidase-activity (b) assay.


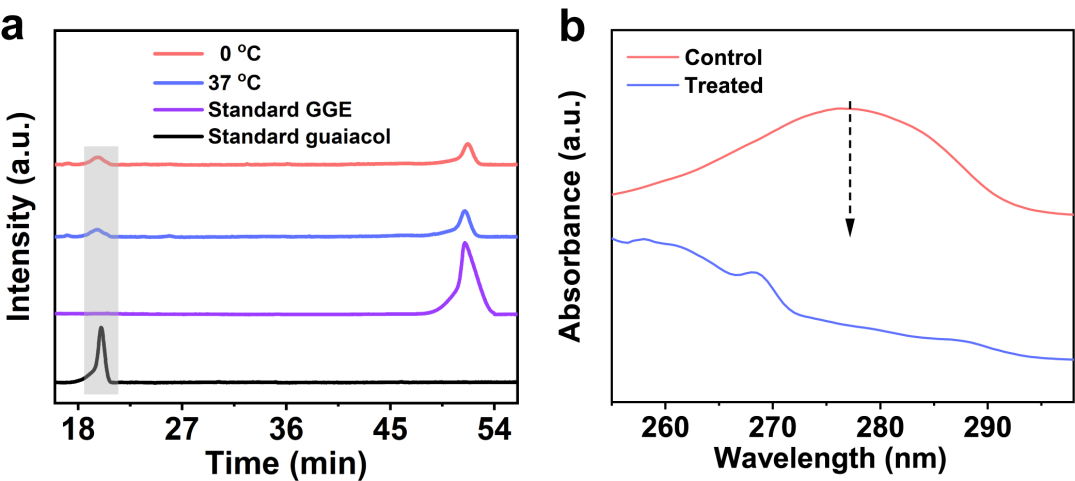


Fig S11. HPLC (a) and UV-vis (b) spectra of GGE in process of treatment.

Table S1 EXAFS fitting parameters of the Mn K-edge and XPS patterns of samples

| Samples | shell | CN | R(Å) | (Mn^3+^+Mn^4+^)% from XPS | Mn ratio (%) from ICP |
| --- | --- | --- | --- | --- | --- |
| D-CuMnO_x_ | Mn-O | 5.8±0.4 | 1.78±0.02 | 71.7 | 50.3% |
|  | Mn-Mn | 5.3±0.2 | 2.48±0.01 |  |  |
| CuMnO_x_ | Mn-O | 5.9±0.2 | 1.9±0.02 | 60.4 | 52.8% |
|  | Mn-Mn | 5.8±0.2 | 2.53±0.01 |  |  |
| Mn_3_O_4_ | Mn-O | 6.0±0.1 | 1.91±0.01 | / | / |
|  | Mn-Mn | 6±0.2 | 2.58±0.01 |  |  |

Table S2 Apparent dynamic parameters of D-CuMnO_x_ towards different substrates (The *K_cat_* and *K_cat_*/*K_m_* were calculated with same mass concentration between D-CuMnO_x_ and CuMnO_x_ for comparison)

| Parameters | | Substrates | |
| --- | --- | --- | --- |
|  |  | TMB | pNPG |
| *K_m_* (mM) | 4 ^o^C | 0.234 | 9.179 |
|  | 37 ^o^C | 0.245 | 7.432 |
| *V*_max_ (μM min^-1^) | 4 ^o^C | 24.54 | 14.4 |
|  | 37 ^o^C | 26.34 | 13.8 |
| *K_cat_* (min^-1^) | 4 ^o^C | 4.09×10^3^ | 0.18×10^3^ |
|  | 37 ^o^C | 4.39×10^3^ | 0.13×10^3^ |
| *K_cat_*/*K_m_* (M min^-1^) | 4 ^o^C | 17.47×10^6^ | 1.85×10^4^ |
|  | 37 ^o^C | 17.91×10^6^ | 1.74×10^4^ |

Table S3 Comparison of GHs-like activity between D-CuMnO_x_ and other reported nanozymes

| Nanozyme | Substrate | Reaction condition | Concentration of nanozyme used | Ref |
| --- | --- | --- | --- | --- |
| Cu_2_O | pNPG | room temperature,  pH 7.0 | 0.01-0.1 mg/mL | ^6^ |
| ZnN_4_-900 | Maltose, isomaltose, cellobiose, sucrose, pNPG | room temperature  pH 6.0-7.0 | 0.1-1 mg/mL | ^7^ |
| Ce-MOF | pNPG | 30 ℃, pH 9.0 | 0.2 mg/mL | ^8^ |
| Cu_3_P and FeP | Maltose, Celluxiose | pH 4.0 | 0.2 mg/mL | ^9^ |
| CuO/SiO_2_ | Cellobiose | 70 ^o^C | 0.025 g in 30 mL | ^10^ |
| This work | pNPG, disaccharide and polysaccharide | -10-50 ^o^C,  pH 3-9 | 0.02-0.1 mg/mL | / |

Table S4 DFT model for the hydrolysis reaction on CuMnO_x_

| Reaction step | Model |
| --- | --- |
| IM0 | 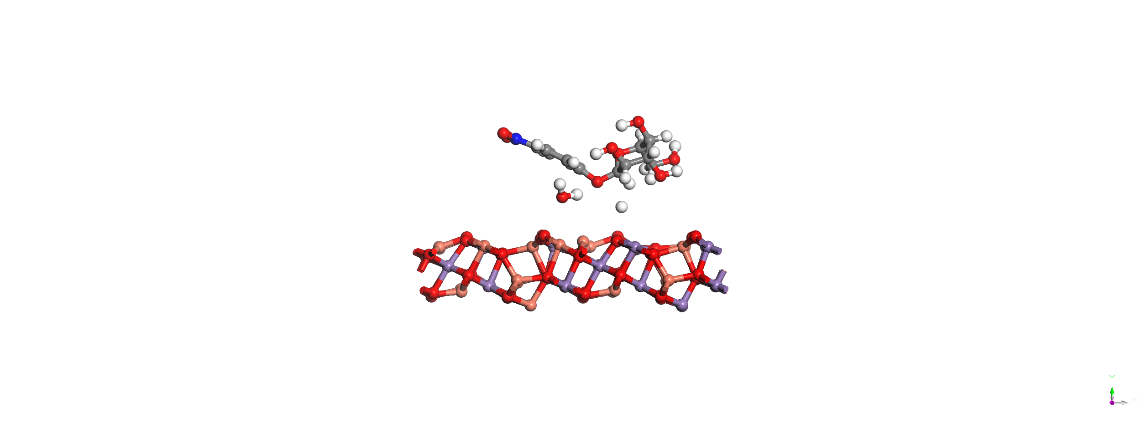 |
| TS1 | 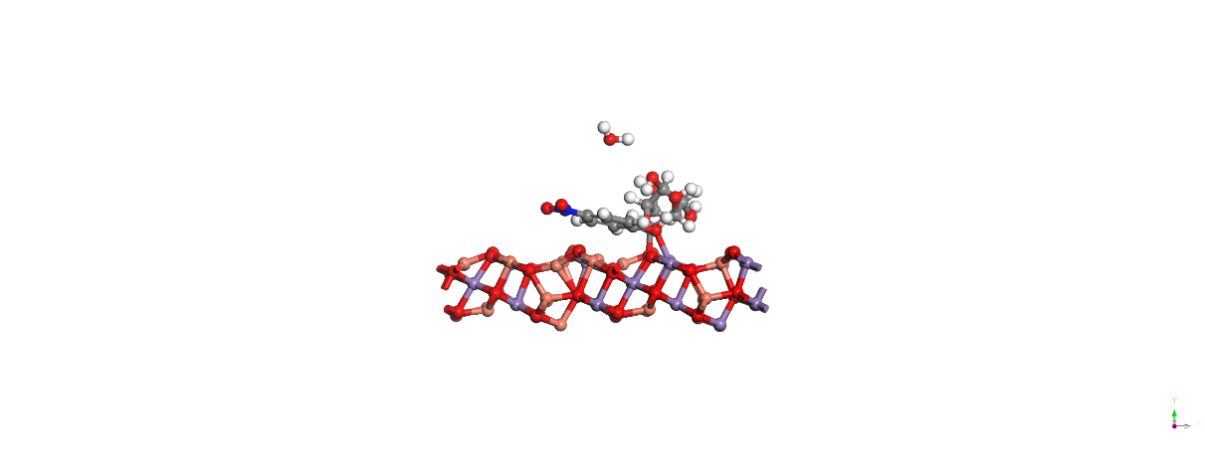 |
| IM1 | 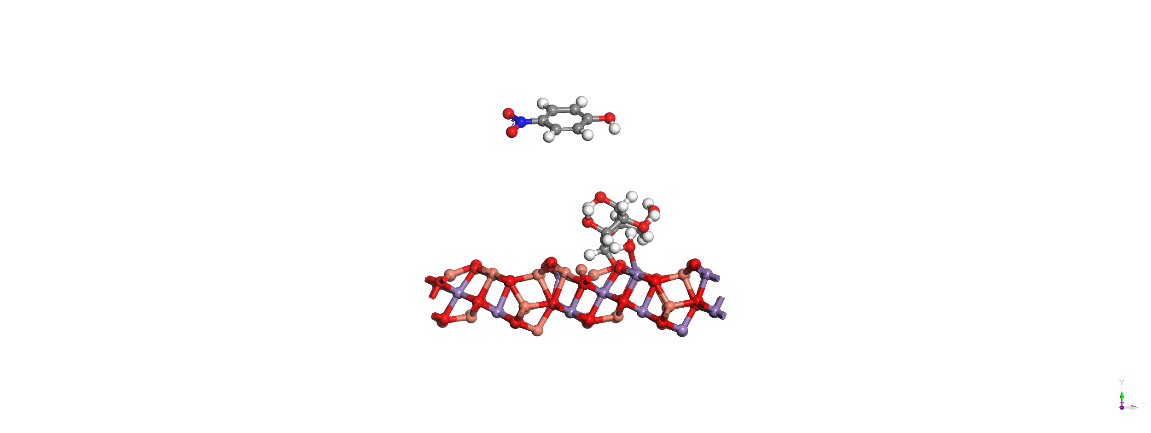 |
| TS2 | 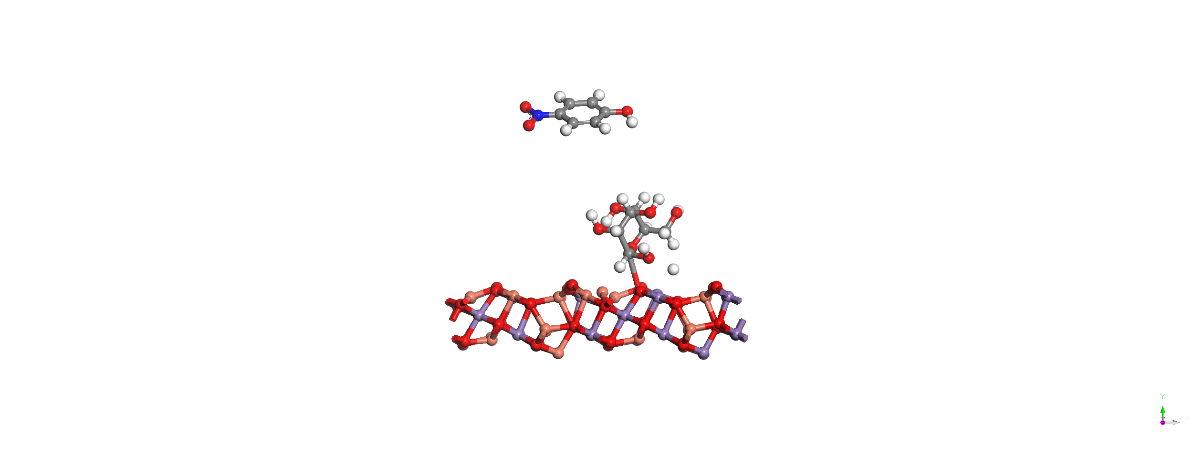 |
| IM2 | 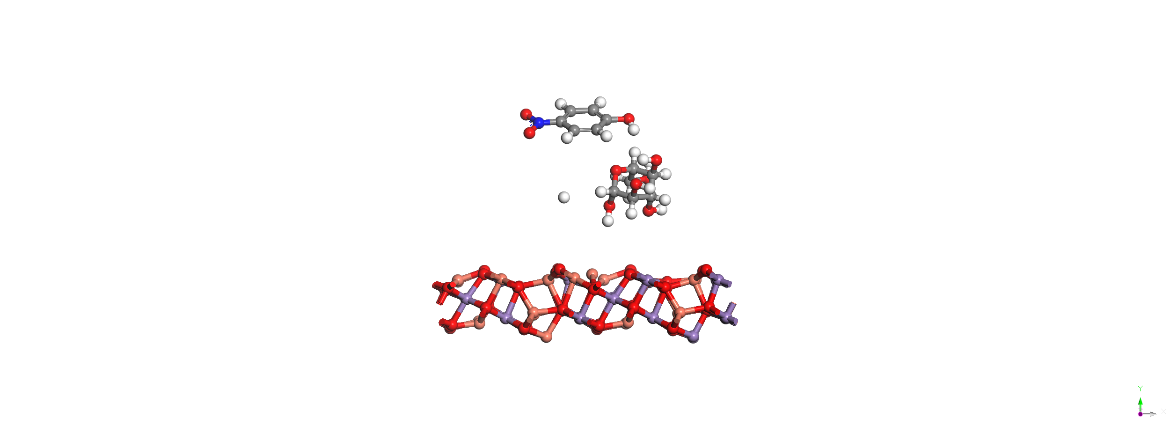 |

Table S5 Comparison of the main products of corn stalk after degradation.

| **No** | **Product** | **0 ^o^C** | **37 ^o^C** |
| --- | --- | --- | --- |
| 1 | 2-Ethoxyethanol | **+** |  |
| 2 | 3-Hydroxybutanal | **+** | **+** |
| 3 | Malonic acid | **+** | **+** |
| 4 | Ethanol | **+** | **+** |
| 5 | 3-Methyl-2-ketobutyric acid | **+** |  |
| 6 | 3-Methoxy-4-methylheptane |  | **+** |
| 7 | Crotonic acid | **+** | **+** |
| 8 | Oxalic acid | **+** | **+** |
| 9 | Ethyl carbamate | **+** |  |
| 10 | 2-Hydroxyethyl carbamate |  | **+** |
| 11 | Ethylene glycol | **+** | **+** |
| 12 | Lactic acid | **+** | **+** |
| 13 | 1,4-Dioxane-2,3-diol | **+** | **+** |
| 14 | Diethylene glycol monoethyl ether acetate |  | **+** |
| 15 | 2-Ethylbutyl Acetate | **+** | **+** |
| 16 | 5-Methyl-2-Isopropylphenol | **+** | **+** |
| 17 | 5-Methylsalicylic acid | **+** |  |
| 18 | 2,6-Dimethoxybenzene-1,4-diol | **+** | **+** |
| 19 | Homovanillic acid | **+** |  |
| 20 | 4-Butoxybutan-1-ol | **+** | **+** |
| 21 | 3-Ethoxy-1-propanol |  | **+** |
| 22 | 2-Ethoxyethanol | **+** | **+** |
| 23 | 2-Hydroxybutyric acid | **+** | **+** |
| 24 | 3,4-Dihydroxymandelic acid | **+** |  |
| 25 | Glycerol | **+** | **+** |
| 26 | Ethylene glycol | **+** | **+** |
| 27 | 2-Tert-butyl-6-methylphenol | **+** |  |
| 28 | Diethylene glycol | **+** | **+** |
| 29 | 2,4-Di-tert-butylphenol |  | **+** |
| 30 | 4-Hydroxybenzoic acid | **+** | **+** |
| 31 | Palmitic acid | **+** | **+** |
| 32 | Stearyl alcohol | **+** |  |
| 33 | 2,6-Dihydroxybenzoic acid | **+** |  |
| 34 | Stearic acid | **+** | **+** |
| 35 | 3-Methylsalicylic acid | **+** |  |
| 36 | Pyridoxine | **+** | **+** |
| 37 | Bis(2-ethylhexyl) phthalate |  | **+** |
| 38 | Isopropyl myristate | **+** | **+** |

Table S6 Mass and carbon efficiency of corn stalk in D-CuMnO_x_-catalytic system.

| Component | Carbon (mg) | | Mass (mg) | |
| --- | --- | --- | --- | --- |
|  | 37 ^o^C | 4 ^o^C | 37 ^o^C | 4 ^o^C |
| Raw corn stalk | 41.4 | 41.4 | 100 | 100 |
| Liquid product | 17.13 | 10.29 | 34.85 | 29.74 |
| Solid residue | 38.6 | 40.3 | 58.32 | 65.81 |
| Carbon efficiency (%) | 30.03 | 24.8 | / | / |
| Mass efficiency (%) | / | / | 93.17 | 95.55 |

Table S7 The mass change of three main components in corn stalk after treatment.

| Component | Raw corn stalk (mg) | Solid residue | | | |
| --- | --- | --- | --- | --- | --- |
|  |  | 37 ^o^C | | 4 ^o^C | |
|  |  | Mass (mg) | Degradation rate (%) | Mass (mg) | Degradation rate (%) |
| Lignin | 19.66 | 9.01 | 54.2 | 10.17 | 48.23 |
| Cellulose | 38.53 | 24.8 | 35.64 | 30.72 | 20.27 |
| Hemicellulose | 26.89 | 15.86 | 40.12 | 16.51 | 38.61 |

**Reference**

1. Cheng, Q.; Yang, Z.; Li, Y., et al., Amorphous/crystalline Cu1.5Mn1.5O4 with rich oxygen vacancies for efficiently photothermocatalytic mineralization of toluene. [*J*]. *Chem. Eng. J* **2023,** *471*, 144295.

2. Shan, C.; Zhang, Y.; Zhao, Q., et al., Acid etching-induced in situ growth of λ-MnO_2_ over CoMn spinel for low-temperature volatile organic compound pxidation. [*J*]. *Environ. Sci.& Technol* **2022,** *56* (14), 10381-10390.

3. Vanderbilt, D., Soft self-consistent pseudopotentials in a generalized eigenvalue formalism. [*J*]. *Phys. Rev. B, Condens. Matter* **1990,** *41* (11), 7892-7895.

4. John P. Perdew, K.B., Matthias Ernzerhof, Generalized gradient approximation made simple. [*J*]. *Phys. Rev. Lett***1996,** *77* (18), 3865-3868.

5. Song, L.; Liu, W.; Xin, F., et al., Study of adhesion properties and mechanism of sodium silicate binder reinforced with silicate fume. [*J*]. *Intern. J. Adhesion Adhesive* **2021,** *106*, 102820.

6. Yu, Z.; Chen, J.; Chao, D., et al., Study on hydrolase mechanism of copper compound nanoparticles and its application in the evaluation of gut bacteria in aquatic environment. [*J*]. *Appl. Catal. B: Environ* **2023,** *330*, 122639.

7. Qiao, C.; Wang, C.; Luo, H., et al., Development of a Zn‐based single‐atom nanozyme for efficient hydrolysis of glycosidic bonds. [*J*]. *Small* **2024,** *20* (46).

8. Liu, S.; Zhang, W.; Wang, H., et al., Revealing moderate Lewis acidity of cerium enhancing hydrolytic activity of MOF‐based nanozymes. [*J*]. *Small* **2025,** *21* (25).

9. Chao, D.; Yu, Z.; Chen, J., et al., A revisiting of transition metal phosphide (Cu3P and FeP) nanozymes for two sugar-related reactions. [*J*]. *Nano Res* **2022,** *16* (1), 189-194.

10. Gu, F.; Liu, H., Hydroxyl radicals-mediated oxidative cleavage of the glycosidic bond in cellobiose by copper catalysts and its application to low-temperature depolymerization of cellulose. [*J*]. *Chinese J Catal* **2020,** *41* (7), 1073-1080.
